# Perivascular adipose tissue‐derived stromal cells contribute to vascular remodeling during aging

**DOI:** 10.1111/acel.12969

**Published:** 2019-05-14

**Authors:** Xiao‐Xi Pan, Cheng‐Chao Ruan, Xiu‐Ying Liu, Ling‐Ran Kong, Yu Ma, Qi‐Hong Wu, Hai‐Qing Li, Yan‐Jun Sun, An‐Qing Chen, Qiang Zhao, Fang Wu, Xiu‐Jie Wang, Ji‐Guang Wang, Ding‐Liang Zhu, Ping‐Jin Gao

**Affiliations:** ^1^ Department of Hypertension, State Key Laboratory of Medical Genomics, Shanghai Key Laboratory of Hypertension Shanghai Institute of Hypertension, Ruijin Hospital, Shanghai Jiao Tong University School of Medicine Shanghai China; ^2^ Key Laboratory of Genetic Network Biology, Institute of Genetics and Developmental Biology Chinese Academy of Sciences Beijing China; ^3^ Department of Cardiac Surgery Ruijin Hospital, Shanghai Jiao Tong University School of Medicine Shanghai China; ^4^ Department of Geriatrics Ruijin Hospital, Shanghai Jiao Tong University School of Medicine Shanghai China

**Keywords:** adipocytes, aging, neointimal hyperplasia, perivascular adipose tissue, perivascular adipose tissue‐derived stromal cells, peroxisome proliferator‐activated receptor‐γ coactivator‐1 α

## Abstract

Aging is an independent risk factor for vascular diseases. Perivascular adipose tissue (PVAT), an active component of the vasculature, contributes to vascular dysfunction during aging. Identification of underlying cell types and their changes during aging may provide meaningful insights regarding the clinical relevance of aging‐related vascular diseases. Here, we take advantage of single‐cell RNA sequence to characterize the resident stromal cells in the PVAT (PVASCs) and identified different clusters between young and aged PVASCs. Bioinformatics analysis revealed decreased endothelial and brown adipogenic differentiation capacities of PVASCs during aging, which contributed to neointimal hyperplasia after perivascular delivery to ligated carotid arteries. Mechanistically, in vitro and in vivo studies both suggested that aging‐induced loss of peroxisome proliferator‐activated receptor‐γ coactivator‐1 α (PGC1α) was a key regulator of decreased brown adipogenic differentiation in senescent PVASCs. We further demonstrated the existence of human PVASCs (hPVASCs) and overexpression of PGC1α improved hPVASC delivery‐induced vascular remodeling. Our finding emphasizes that differentiation capacities of PVASCs alter during aging and loss of PGC1α in aged PVASCs contributes to vascular remodeling via decreased brown adipogenic differentiation.

## 1 INTRODUCTION

Aging of the population is one of the major challenges facing public health systems. Vascular diseases increase with age, even in populations without other major risk factors.(Lakatta & Levy, 2003) Endothelial inflammation, reduced nitro oxide bioavailability, and activation of smooth muscle cell (SMC) have been documented to be involved in the process of vascular senescence, which is characterized by increased arterial stiffness and vascular fibrosis.(Pierce, Lesniewski, Lawson, Beske, & Seals, 2009; Wang, Monticone, & Lakatta, 2010) Perivascular adipose tissue (PVAT) is situated outside the adventitial layer and surrounds most of the systemic blood vessels.(Rajsheker et al., 2010) Aging promotes superoxide production and adipocyte dysfunction in the PVAT, which subsequently contributes to aging‐related vascular injury.(Fleenor et al., 2014; Gomez‐Serrano et al., 2017; Xu et al., 2018) In addition to adipocytes, PVAT contains macrophages, leukocytes, as well as stromal cells and autonomic nerves.(Brown et al., 2014; Ruan et al., 2015) However, little is known about the effects of aging on the other cells in the PVAT and their roles in aging‐related vascular diseases.

Adipose tissue is rich in pluripotent adipose‐derived stromal cells (ASCs). These cells express the surface markers of adult mesenchymal stem cell. ASCs have the potential to differentiate into multiple lineages of cells, such as endothelial cell, smooth muscle cell, osteoblast, and adipocyte, under specific culture conditions.(Bhumiratana et al., 2016; Rivera‐Gonzalez et al., 2016; Silva et al., 2014) However, no attention has been given to characterizing the resident stromal cells in the PVAT, and the contribution of PVAT‐derived stromal cells (PVASCs) to vascular homeostasis is still unclear. It is well documented that senescence‐related decline in pluripotent stromal cell function contributes to aging‐associated impairments in tissue regeneration.(Alt et al., 2012; Goodell & Rando, 2015; Hassanpour, Cheraghi, Siavashi, Rahbarghazi, & Nouri, 2016) Therefore, we hypothesized that aging‐induced PVASCs dysfunction and the altered differentiation capability contribute to vascular remodeling.

In this study, we utilized single‐cell RNA sequencing (scRNA‐seq) to identify the transcriptional characteristics of resident PVASCs in the PVAT of young and aged mice. We identified different clusters of PVASCs between young and aged mice and revealed a transcriptional heterogeneity associated with altered differentiation potential of PVASCs during aging. Mechanistically, aging‐induced loss of peroxisome proliferator‐activated receptor‐γ coactivator‐1 α (PGC1α) was a key regulator of decreased brown adipogenic differentiation in senescent PVASCs, which accelerated neointimal hyperplasia after perivascular delivery to injured arteries. More importantly, we also demonstrated the existence of human PVASCs (hPVASCs) by utilizing samples from patients that underwent coronary artery bypass grafting (CABG) surgery. Overexpression of PGC1α improved hPVASC delivery‐induced vascular fibrosis after vascular injury in nude mice.

## 2 RESULTS

### 2.1 Identification of resident stromal cells in the PVAT of young and aged mice

**Figure 1:**
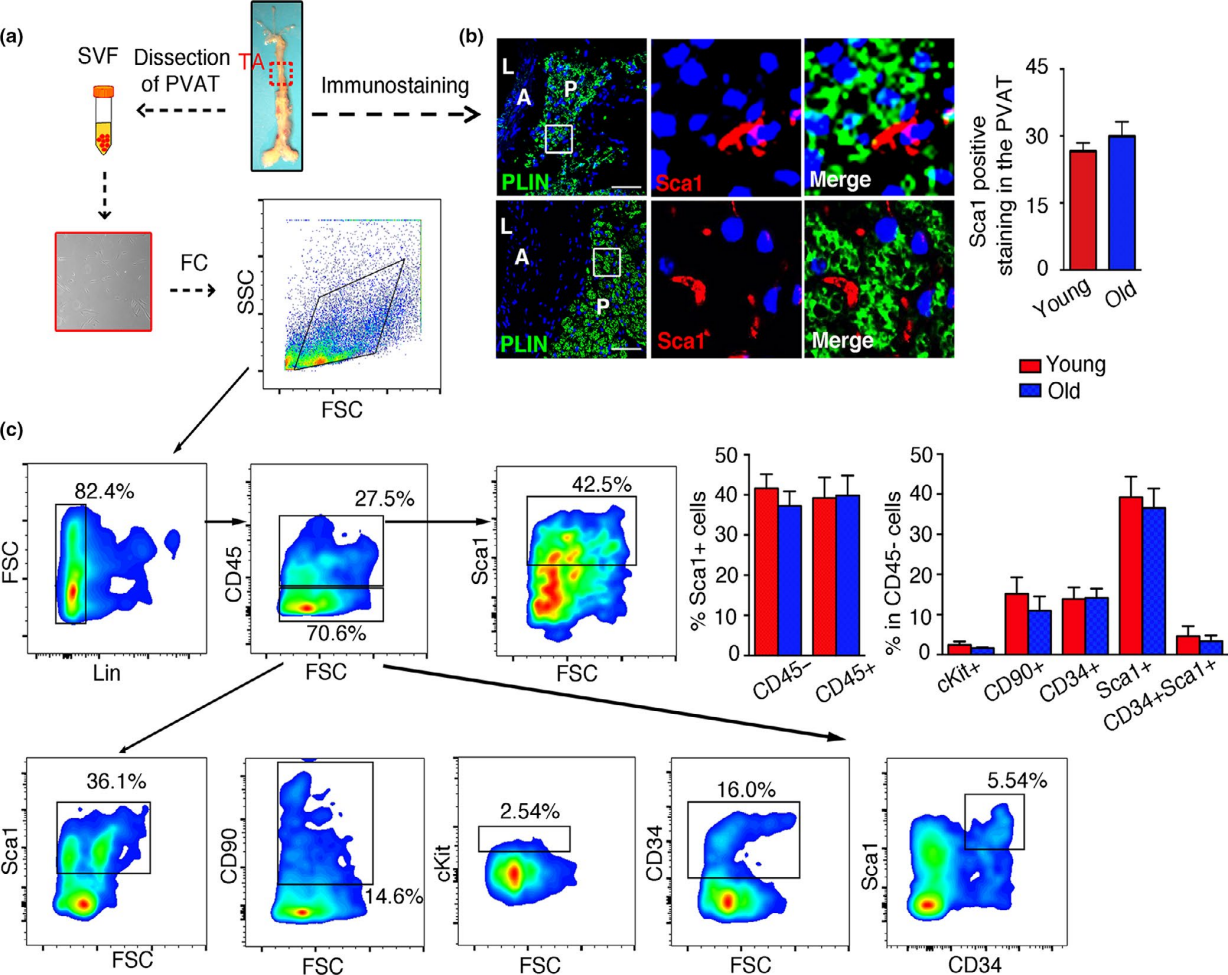
Characterization of resident stromal cells in the perivascular adipose tissue (PVAT) in 3‐, or 16‐ to 18‐month‐old mice. (a) The thoracic aortas (TA) were dissected from young or old mice for immunostaining, cell culture, and flow cytometric analysis. (b) The TA was immunostained for adipocytes marker (PLIN) and Sca1. A represented aorta, L represented lumen, and P represented PVAT. Scale bar 100 μm. (c) Representative flow cytometry density plot showed the expression of cKit, CD90, CD34, and Sca1 on gated Lin‐CD45+ cells, and Sca1+ cells gated from Lin‐CD45‐ cells in the stromal vascular fraction of PVAT. *n* = 5 per group

To detect the resident PVASC in the PVAT, the thoracic aortas of 3‐, or 18‐ to 20‐month‐old mice were carefully dissected for immunostaining (Figure 1a). Sca1‐positive stromal cells resided within the PVAT (PLIN^+^adipocytes), and there was no significant difference between young and aged mice (Figure 1b). Next, the immunophenotypic profiles of PVASCs were detected by flow cytometric analysis. Both young and old Lin^−^ PVASCs showed comparative Sca1^+^CD45^+^ macrophage progenitor cells and Sca1^+^CD45^−^ stem cells. Lin^‐^CD45^−^ cells showed positive staining for mesenchymal stem cell (MSC) markers Sca1 (>40%), CD90 (≈15%), and little cKit expression (<5%). Besides, Lin^−^CD45^−^ cells included a small amount of endothelial progenitor cells (CD34^+^Sca1^+^, ≈5%). These had no significant difference in young and aged mice (Figure 1c). PVASCs obtained from digested PVAT of young mice were cultured to passage 3 and were further characterized by flow cytometry for MSC markers, and their multiple differentiation capacity in vitro by treating cells with specific differentiation induction media. Flow cytometric analysis detected MSC marker expression, including Sca1, CD90, CD146, and CD29 (>80%), in these cells (Figure S1 a, b). Specific staining and immunostaining revealed that these cells expressed Sca1 and were capable of differentiating into endothelial cells (ECs), smooth muscle cells (SMCs), osteoblasts, and adipocytes (Figure S1 c‐g). Their differentiation capacity was further verified by qPCR analysis (Figure S1 h‐k). The above data indicate that these cells residing in the PVAT are multipotent.

We next determined the effects of aging on surface markers in old PVASCs. Interestingly, there was no significant morphological difference between the young and old PVASCs in the bright field (Figure S2 a). The staining of MSC markers (Sca1 and CD90) was similar between the two groups (Figure S2 b‐c). Flow cytometric analysis showed that CD29 (>75%), CD105 (>65%), CD90 (>60%), Sca1 (>85%), CD44 (>25%), and CD146 (>35%) had no significant difference between the two groups. And both stromal cells showed a little positive staining for endothelial progenitor marker (CD34, <8%) and endothelial marker (CD31, <5%) (Figure S2 d‐e).

### 2.2 ScRNA‐seq reveals an altered differentiation capacity of PVASCs during aging

**Figure 2:**
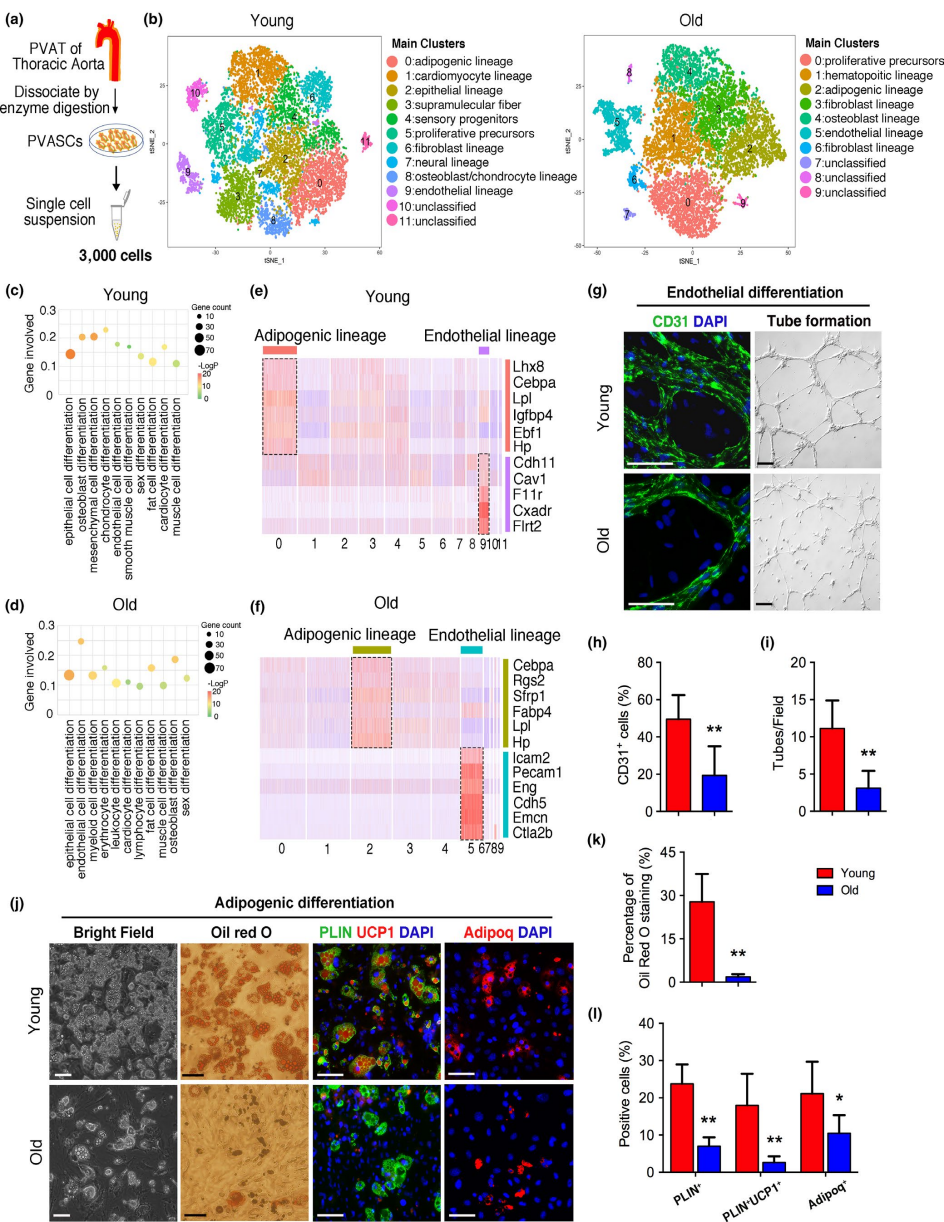
Single‐cell RNA sequencing (scRNA‐seq) reveals the transcriptional heterogeneity in young and old perivascular adipose tissue‐derived stromal cells (PVASCs). (a) Experimental workflow. PVAT was dissected from aortas of young or old mice and dissociated by enzymatic digestion for scRNA‐seq. (b) t‐SNE plot of scRNA‐seq data from young or old PVASCs. (c, d) Gene ontology analysis of differentiation process. (e, f) Heat map of average expression for representative genes indicative of adipogenic lineage and endothelial lineage and cell‐type classification of the main clusters from young (e) and old (f) data set. (g‐l) The adipogenic and endothelial differentiation were determined by specific staining in the young and old PVASCs. Scale bar 50 μm. *n* = 5 independent experiments

We next performed scRNA‐seq assay to reveal the transcriptional characteristics of young and old PVASCs. To avoid any bias associated with the use of cell surface markers that may be differentially expressed on PVASCs, 3,000 young and 3,000 old primary cultured PVASCs were randomly collected and included in the scRNA‐seq analysis. Clustering young PVASCs produced 12 main distinct populations, while old PVASCs produced 10 populations (Figure 2a, Figure S3a, b and Table S1). To evaluate the potential functional heterogeneity of young and old PVASCs, we performed gene ontology (GO) analysis. It revealed that young and old PVASCs both were enriched for vasculature development, which suggested it play a critical role in vascular remodeling. Additionally, young PVASCs seemed to be enriched for negative regulation of VSMC proliferation which not found in old group, while old PVASCs that more genes involved in oxide metabolic process (Figure S3 c‐f). We systematically analyzed the differential expression of marker genes for each cluster and defined 10 putative cell types (cluster 0–9) in young PVASCs and 7 (cluster 0–6) in old PVASCs, while several small clusters remained unclassified (Figure 2b). It showed much more diversity in young rather than old PVASCs, including adipogenic, cardiomyocyte, epithelial, neural, and endothelial lineage, as well as sensory progenitors and supramolecular fiber. Besides adipogenic, endothelial, and osteoblast lineage, the old PVASCs owned a cell type of hematopoietic lineage. GO analysis further indicated the altered differentiation potential of aging PVASCs, in which muscle cell differentiation, adipocyte differentiation, and EC differentiation were closely linked to vascular remodeling (Figure 2c, d). Among these putative cell‐type populations, two major clusters of genes are significantly related to adipogenic and endothelial differentiation. In the young PVASCs, cells in cluster 0 and cluster 9 expressed, respectively, genes associated with adipogenic and endothelial differentiation (Figure 2e and Figure S4 a). Similarly, the cluster 2 in the old PVASCs is enriched in the gene ontology terms related to adipogenic differentiation, whereas genes enriched in cluster 5 indicate structural changes toward an endothelial fate (Figure 2f and Figure S4 b). In addition, cellular signaling pathways associated with adipogenic differentiation, including BMP and MAPK signaling pathway that promote adipogenesis, were upregulated in young data sets (Figure S4 c, d). These suggest an altered differentiation heterogeneity of old PVASCs compared to young cells.

To demonstrate the effects of aging on PVASC function and differentiation, young and old PVASCs were examined for proliferation and migration assay and then differentiated into ECs, SMCs, osteoblasts, and adipocytes, Firstly, the proliferation (Edu‐positive cells) and migration had no significant difference between young and old PVASCs (Figure S5). Indeed, old PVASCs showed a reduced endothelial differentiation (sparse tubular structures and reduced CD31 staining compared with young; Figure 2g‐i), as well as decreased brown adipogenic differentiation (reduced intracellular lipid droplet, oil red o staining, PLIN, UCP1, and Adipoq‐positive cells compared with young; Figure 2j‐l). These were further confirmed by decreased EC and brown adipocyte marker gene expression (CD31, Vecad, Ucp1, and Pparg; Figure S6). In contrast, old and young PVASCs had similar differentiation capacity toward SMCs and osteoblasts (Figure S7). These suggest a decreased endothelial and brown adipogenic differentiation capacity of PVASCs during aging.

**Figure 3:**
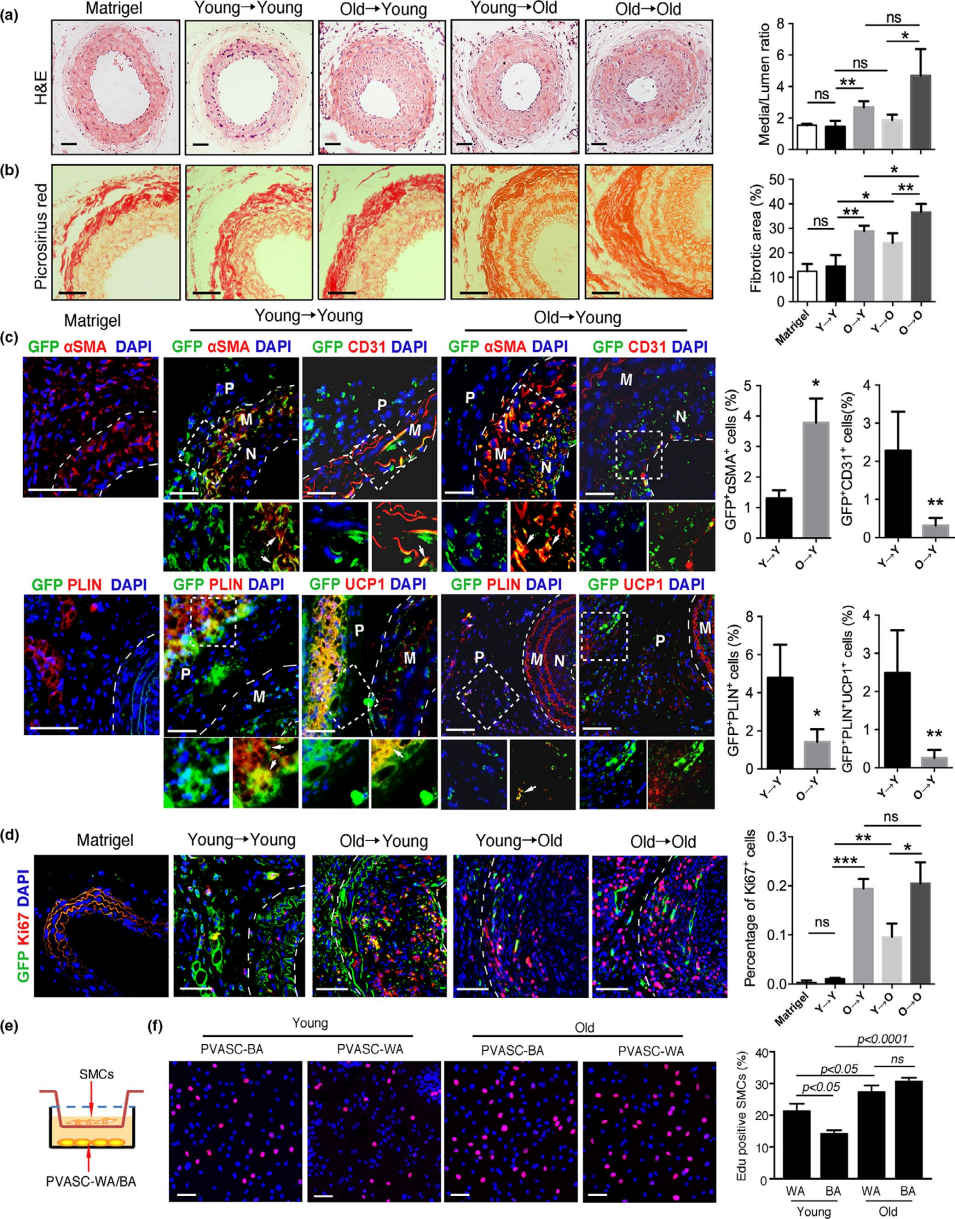
The altered differentiation of old perivascular adipose tissue‐derived stromal cells (PVASCs) accelerates neointimal hyperplasia after carotid ligation injury. (a, b) Left: H&E and picrosirius red‐stained sections of carotid arteries. Right: quantitative analysis of medium–lumen ratio and arterial fibrotic area of injured arteries. *p < 0.05. Scale bar 50 μm. *n* = 6 per group. (c) The carotid arteries sections were co‐stained for GFP and CD31, GFP and SMA, GFP and PLIN, GFP and UCP1. (d) Representative images showed the proliferating cells (Ki67 positive). (e) Schematic of the Transwell coculture chamber. PVASC‐WA indicated PVASCs‐derived white adipocytes. PVASC‐BA indicated PVASCs‐derived brown adipocytes. (f) The proliferation of SMCs cocultured with young or old PVASC‐WA/BA was measured by Edu staining. Red indicated positive staining for Edu. *n* = 4 independent experiments

We next determined the pathological role of aged PVASCs in the vascular remodeling process. Young or old PVASCs isolated from GFP‐transgenic mice embedded in the Matrigel were implanted in the perivascular tissue of carotid arties of young or old mice after ligation injury, Matrigel‐only delivery as the control group. As shown in Figure 3a‐b, both in young and in old recipient mice, old PVASC delivery promoted neointimal hyperplasia and perivascular collagen deposition compared to young cells. Furthermore, the degree of arterial fibrosis increased in old recipient rather than the young, but there was no significant difference in neointimal hyperplasia. Immunostaining indicated that in the young recipient mice, young GFP^+^ cells differentiated into ECs (positive for CD31 staining) or myofibroblasts (positive for αSMA staining) in the neointima. Interestingly, these cells also differentiated into brown adipocytes (positive both for PLIN and for UCP1 staining) in the perivascular tissue. In contrast, old GFP^+^ cells showed an increased staining for αSMA and a decreased staining for CD31 in the neointima. In the perivascular tissue, PLIN‐ and UCP1‐positive brown adipocytes could not be detected in old GFP^+^ PVASCs (Figure 3c). When young or old PVASCs were implanted to old recipients, the results were similar to the young recipients, but young PVASCs exposed to the aged environment loss the potential to differentiate into brown adipocytes (Figure S8). More importantly, perivascular delivery of old PVASCs promoted recipient local vascular cell proliferation, and aged environment seems to contribute to proliferation of vascular cells (Figure 3d). In vitro coculture experiment further demonstrated that young PVASC‐derived brown adipocytes inhibited SMC proliferation, while old PVASC‐derived adipocytes promoted SMC proliferation (Figure 3e, f). To further demonstrate the role of brown adipocytes in SMC proliferation, we transplanted brown adipose tissue (BAT) or white adipose tissue (WAT) into injured carotid artery. The results showed that BAT rather than WAT inhibited SMC proliferation in recipient mice (Figure S9). These together suggest that aged PVASCs show decreased brown adipocyte differentiation capacity and accelerate neointimal hyperplasia.

### 2.3 The differentiation of PVASC to adipocyte is dependent on PGC1α

**Figure 4:**
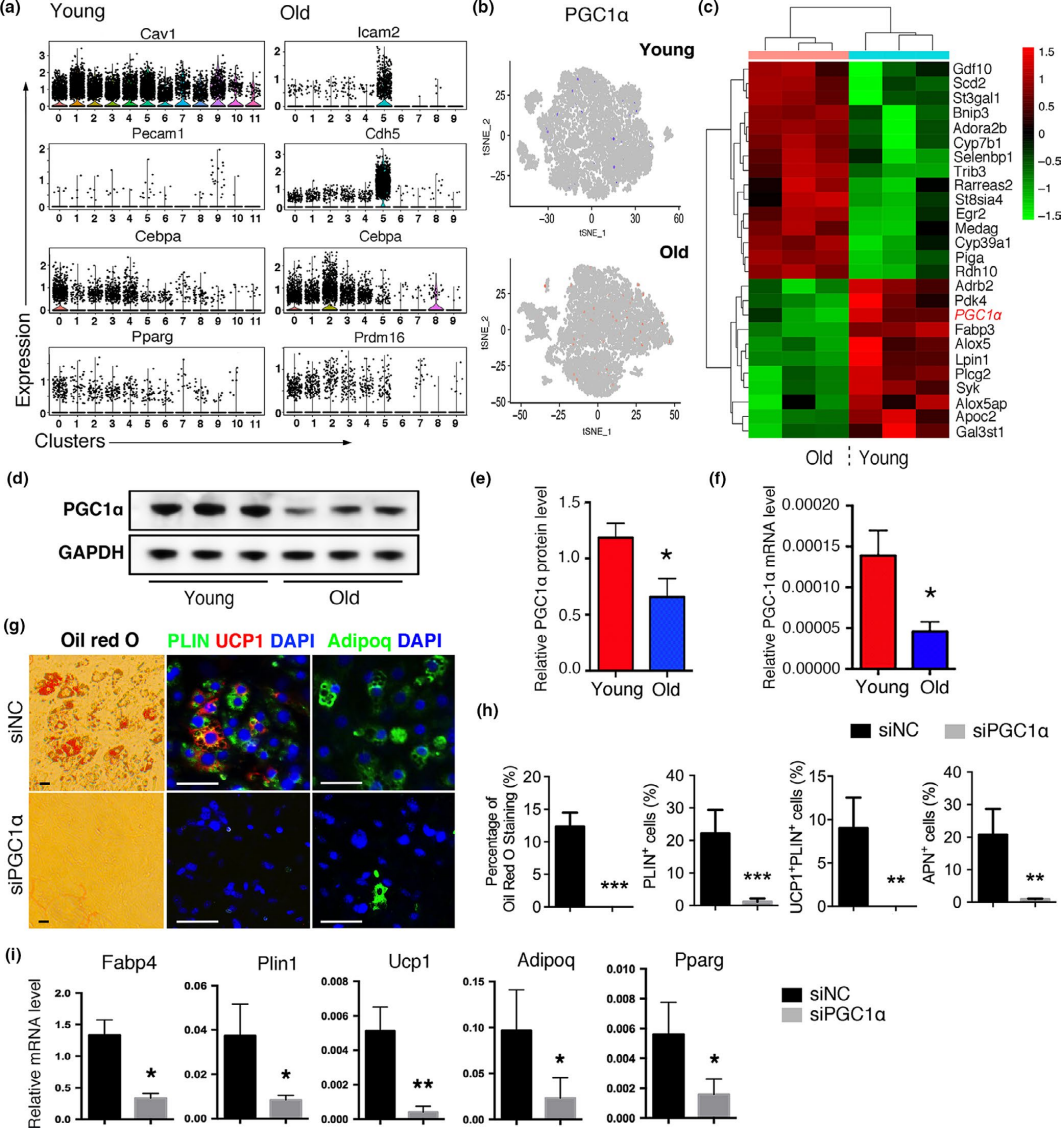
Aging induces downregulation of PGC1α in the PVASCs. (a) Violin plots showing the expression of selected genes in cell‐type clusters. (b) t‐SNE plots depicting the expression of PGC1α in young and old PVASCs. (c) Heat map showed different gene expression profiling between young and old PVASCs. PGC1α was red font. (d, e) Representative Western blot and quantitation for PGC1α in PVASCs of young and old mice. (f) The expression of PGC1α was determined by qPCR analysis. *p < 0.05 versus young. *n* = 4 independent experiments. (g, h) The adipogenic differentiation was determined by specific staining in PVASCs infected with lentivirus containing PGC1α‐short hairpin RNA (siPGC1α) or negative control (siNC). Scale bar 50 μm. (i) The adipogenic differentiation was determined by qPCR of specific markers. *p < 0.05, **p < 0.01 versus siNC. *n* = 5 independent experiments

To understand the molecular mechanism that underlies the distinct adipogenic differentiation of old PVASCs, we further analyzed marker genes that are indicative of adipogenic and endothelial lineages in all clusters. The young endothelial cluster showed a high expression of Cav1 and Pecam1, while the old endothelial cluster showed high expression of Icam2 and Cdh5. The young adipogenic cluster had a high expression of Cebpa and Pparg, while the old adipogenic cluster had a high expression of Cebpa and Prdm16 (Figure 4a). Interestingly, brown adipocyte marker gene PGC1α was detected in many clusters of both groups (Figure 4b). CDNA microarray further showed a decreased expression of PGC1α in old PVASCs compared to young cells (Figure 4c). Immunoblot and qPCR verified decreased PGC1α expression in the old PVASCs (Figure 4d‐f).

To determine the role of PGC1α in brown adipogenic differentiation of PVASC, we knocked down PGC1α (siPGC1α) in young and overexpressed PGC1α (LV‐PGC1α) in old PVASCs by utilizing lentivirus. PGC1α‐knock down inhibited brown adipogenic differentiation in the young PVASCs (Figure 4g, h). QPCR and Western blot analysis further demonstrated downregulation of Pparg, Ucp1, Adipoq, Plin1, and Fabp4 expression in siPGC‐1α‐transfected PVASCs (Figure 4i and Figure S10 c). PGC1α‐knock down in young PVASCs also inhibited tube formation and endothelial differentiation. (Figure S10 d) On the other hand, PGC1α‐overexpression rescued the brown adipogenic differentiation capacity of old PVASCs (Figure S11).

**Figure 5:**
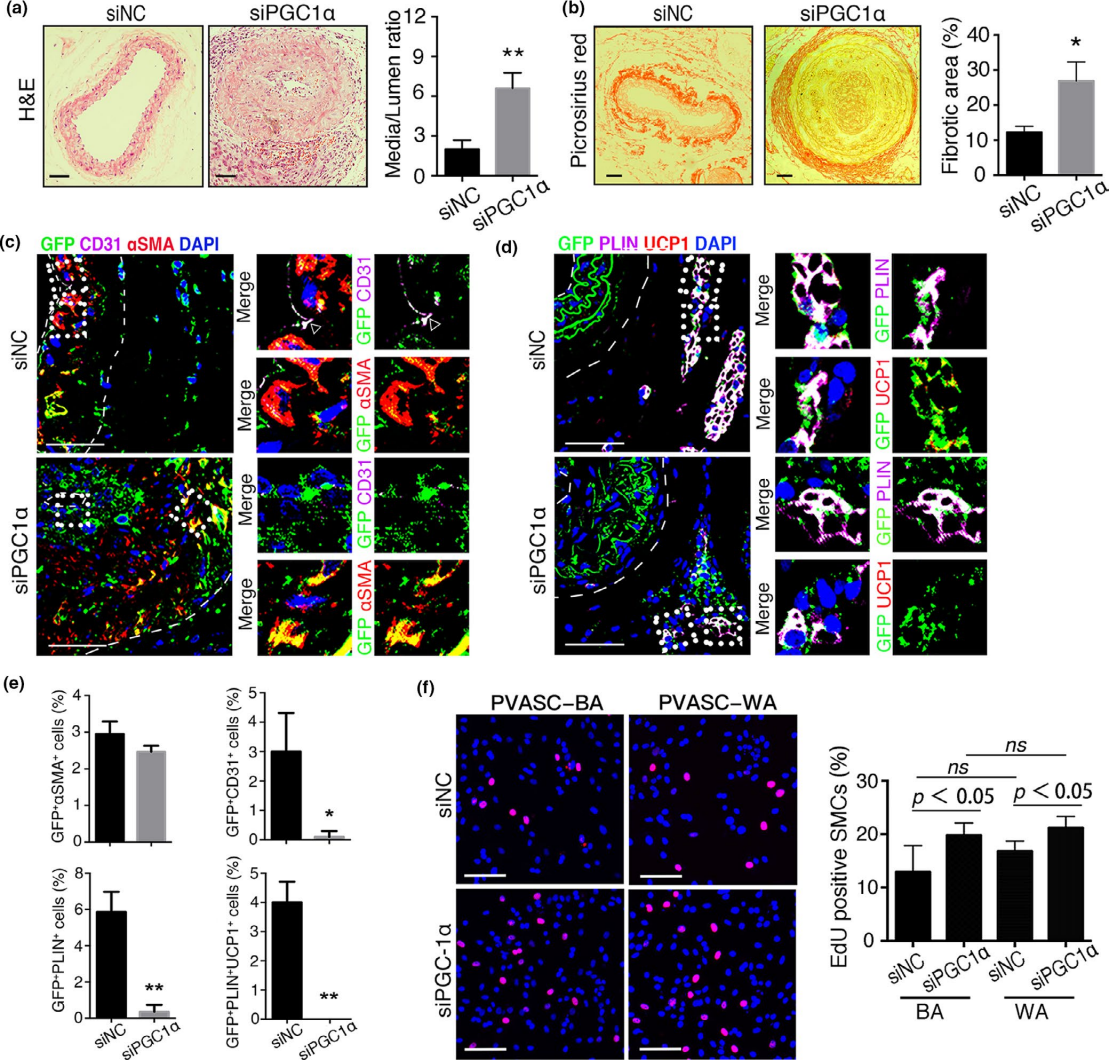
PGC1α‐knockdown in young PVASCs accelerates neointimal hyperplasia after perivascular delivery. (a,b) H&E and picrosirius red‐stained sections of carotid arteries 28 days after PGC1α‐short heparin RNA (siPGC1α) or negative control (siNC) infected PVASCs delivery to perivascular tissue of injured arteries. (c‐e) The carotid arterial sections were co‐stained for GFP, CD31, SMA (c) and GFP, PLIN, UCP1 (d). Scale bar 50 μm. *n* = 5 per group. *p < 0.05, **p < 0.01, versus negative control. (f) The proliferation of SMCs cocultured with siPGC1α or siNC‐PVASC‐WA/BA was measured by Edu staining. Red indicated positive staining for Edu. *n* = 4 independent experiments

We next embedded young PVASCs transfected with lentivirus of siPGC1α or siNC in Matrigel, which were implanted into the perivascular tissue of injured vessels after ligation injury. Delivery of PGC1α‐knockdown GFP^+^ PVASCs exacerbated neointima hyperplasia and perivascular collagen deposition (Figure 5a‐b). Interestingly, GFP^+^ cells transfected with siPGC1α showed an increased αSMA staining, but a decreased CD31 staining in the neointima compared to siNC‐PVASC delivery. Correspondingly, few GFP^+^ cells expressed PLIN and UCP1 (Figure 5c‐e). Moreover, in vitro coculture experiment showed that PGC1α‐knockdown PVASC‐derived adipocyte promoted SMC proliferation (Figure 5f). And VSMCs cocultured with adipocytes derived from siPGC1α‐PVASCs showed upregulation of Col1a1 and Col3a1 (Figure S12). These together suggest that loss of PGC1α plays a critical role in attenuating brown adipocyte differentiation of PVASC during aging, and the altered differentiation contributes to vascular remodeling after vascular injury.

### 2.4 Identification and characterization of human PVASCs

**Figure 6:**
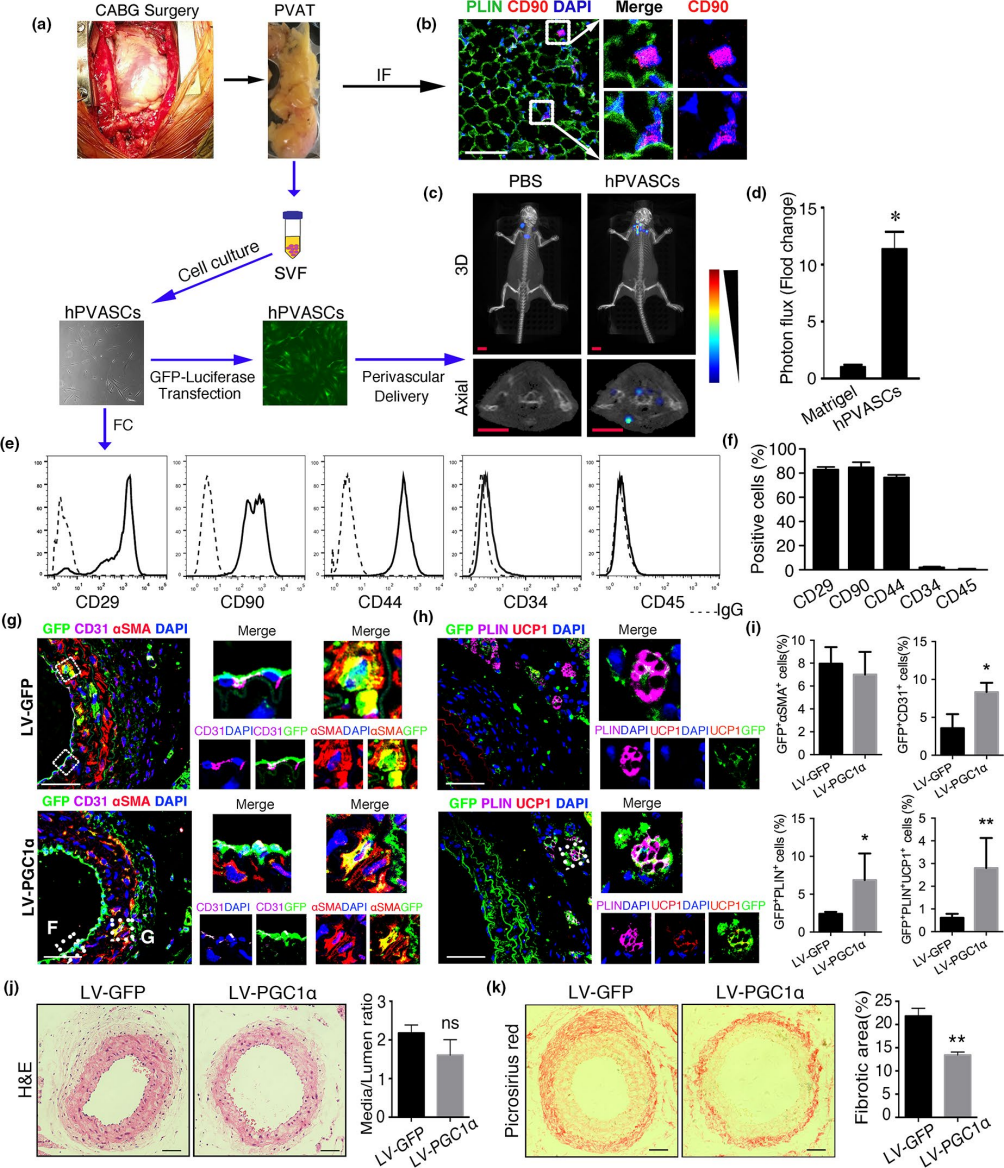
Human perivascular adipose tissue‐derived stromal cells (hPVASCs) contribute to vascular remodeling in nude mice. (a) The diagram of patients' sample collection, hPVASC culture and perivascular delivery to injured arteries in nude mice. (b) Representative images of PVAT immunostaining for adipocyte marker (PLIN) and stem cells marker (CD90). (c) Representative bioluminescent imaging of carotid arteries after Matrigel only or hPVASCs transfected with GFP‐luciferase gene for 1 week. Scale bar 5 mm. (d) Quantitative analysis of photon flux for bioluminescent imaging. (e, f) Flow cytometric analysis of hPVASCs showed positive markers of mesenchymal stem cell and negative markers of hematopoietic and endothelial cell. (g‐i) The carotid arteries sections with implanted hPVASCs that infected with control vector (LV‐GFP) or PGC1α‐overexpressed(LV‐PGC1α) lentivirus were co‐stained for GFP, CD31, and SMA or co‐stained for GFP, PLIN, and UCP1 after hPVASCs delivery in injured arteries. Scale bar 50 μm. *p < 0.05, **p < 0.01, versus LV‐GFP. *n* = 5 per group. (j, k) Representative light micrographs and quantitative analysis of H&E and Picrosirius red‐stained sections of injured arteries

To test characteristics of PVASCs in the PVAT of human blood vessels, we obtained the PVAT from patients (average 63 years old) underwent CABG surgery (Figure 6a). Immunostaining showed that CD90‐positive stromal cells were present among the PLIN+ adipocytes in the adipose tissue (Figure 6b). The cultured hPVASCs showed positive MSC markers CD29 (>80%), CD90 (>80%), and CD44 (>75%), and negative for EPC marker CD34 (<6%) and hematopoietic marker CD45 (<2%) (Figure 6e‐f). These cells were further identified by their multiple differentiation toward ECs, SMCs, osteoblasts, and adipocytes (Figure S13).

Next, to investigate the in vivo fate of these hPVASCs within the vascular remodeling process, the nude mice that underwent carotid arteries ligation injury were used as recipient for hPVASC implantation. The hPVASCs transfected with GFP–luciferase lentivirus were embedded in Matrigel, which were implanted in the perivascular tissue of injured vessels. As shown in Figure 6c‐d, the significant bioluminescent signals around the left common carotid arterial area indicated successful implantation of hPVASCs in nude mice. H&E and picrosirius red staining showed that hPVASCs delivery promoted neointima hyperplasia and perivascular collagen deposition compared with Matrigel only (Figure S14).

In accordance with the in vivo fate of PVASCs from mice PVAT, hPVASCs could differentiate into myofibroblasts and ECs in the neointima, as well as adipocytes in the PVAT, but UCP1‐positive brown adipocytes could not be detected. In contrast, hPVASCs that overexpressed PGC1α showed an increased portion of PLIN and UCP1‐positive brown adipocytes (Figure 6g‐i). Moreover, PGC1α‐overexpression attenuated perivascular collagen deposition, although it did not significantly affect neointimal hyperplasia in nude mice after ligation injury (Figure 6j, k). These provide direct evidence that the hPVASCs may be involved in the vascular remodeling process via spontaneously differentiating into vascular cells and perivascular brown adipocytes during aging.

## 3 DISCUSSION

Previous studies have demonstrated that PVAT participates in the regulation of neointimal formation, vascular fibrosis, and vasomotor reaction through releasing adipokines, inflammatory factors, and other vasoactive substances.(Antonopoulos et al., 2015; Chatterjee et al., 2009; Greenstein et al., 2009; Margaritis et al., 2013; Withers et al., 2014) Herein, the first direct identification of stromal cells within PVAT further extends our understanding of the vascular surrounding tissues. The scRNA‐seq data reveal the differentiated heterogeneity of these PVASCs. More importantly, aged PVASCs show a different transcriptional profile in scRNA‐seq which suggests an altered differentiation potential. Mechanically, aging induces a decreased PGC1α expression, which results in reduced brown adipogenic differentiation of PVASCs. Inhibition of PGC1α in young PVASCs restrains brown adipocyte differentiation and aggravates neointimal hyperplasia after vascular injury.

Aging is an independent risk factor for vascular diseases and is associated with functional, structural, and mechanical changes in arteries independent of disease.(Lakatta & Levy, 2003; Meyer et al., 2016) Previous studies have showed that aging exacerbates neointima lesions after vascular injury through promoting macrophage infiltration and higher sensitivity of SMCs to proliferation stimuli.(Eghbalieh et al., 2012; Rodriguez‐Menocal et al., 2014) Aging induces superoxide production in the PVAT and contributes to arterial stiffness. Herein, our findings show that PVASC transcriptional landscape is unexpectedly diverse between young and aged mice. To our surprise, these are few clusters of scRNA‐seq involved in the regulation of cell senescence in aged PVASCs. Functional tests also showed that there is no significant difference in the migration, proliferation, and ROS production between young and aged PVASCs. It is worth noting that scRNA‐seq suggests an altered differentiation capacity in aged PVASCs compared to the young. In line with the transcriptomic analysis, in vitro and in vivo experiments also indicated that aged PVASCs show a decreased endothelial and brown adipogenic differentiation. In addition, young PVASCs subjected to an aged environment exhibited functional decline, which suggested vascular microenvironment aging plays a critical role in PVASC differentiation. It is worth noting that perivascular adipocytes inhibit SMC proliferation and attenuate neointima lesions after endovascular injury.(Takaoka et al., 2009) The formation of PVASC‐derived brown adipocytes indicates a possibility that PVAT protects against ligation injury‐induced neointima formation through the activation and brown adipogenic differentiation of resident stromal cells.

PVAT‐derived stromal cells have the dual ability to differentiate into both white and brown adipocytes in vitro and in vivo. This ability is especially robust in young PVASCs. In contrast, aged cells show a marked decline in capacity of brown adipocyte differentiation. This suggests an aberrant secretion of adipokines and cytokines from aged PVASC‐derived brown adipocytes, thus blocking their protective effects against vascular injury, which in turn initiates the proliferation and migration of myofibroblasts, ultimately inducing neointima lesions.(Antonopoulos et al., 2017) PVAT is a special adipose tissue, which contains both white and brown adipocytes.(Brown et al., 2014) Brown adipocytes utilize glucose and lipids to generate heat and are associated with improved cardiometabolic health.(Chang et al., 2012; Friederich‐Persson, Cat, & A., Persson, P., Montezano, A.C., and Touyz, R.M., 2017) PVAT undergoing a brown‐to‐white transition may be a driving factor of vascular disease. Restoring the brown phenotype of PVAT could be a potential target for improving vascular injury.(Aldiss et al., 2017; Kiefer, Cohen, & Plutzky, 2012) The brown adipogenic differentiation of PVASCs provides a possibility of rebrowning PVAT except for white‐to‐brown adipocyte transition. It is worth noting that young PVASC‐derived brown adipocytes inhibit SMC proliferation, while aged PVASC‐derived adipocytes promote SMC proliferation. Besides，directly transplanting BAT or WAT to the perivascular area showed that BAT rather than WAT improved neointimal formation after vascular injury. Aged PVASCs via perivascular delivery accelerates recipient myofibroblast proliferation in neointima lesions. These indicate that the paracrine role of PVASC‐derived cells is altered during aging and affects the neointima formation after ligation injury.

PGC1α is a negative regulator of vascular aging. Deficiency of PGC1α promotes a vascular senescence phenotype including mitochondrial abnormalities, reduced telomerase activity, and increased oxidative stress.(Xiong, Patrushev, Forouzandeh, Hilenski, & Alexander, 2015; Xiong et al., 2013) PGC1α also is a central positive regulator of adipocyte browning. It has been well documented that PGC1α promotes mitochondrial respiration and heat production via controlling mitochondrial gene expression in brown adipocytes.(Dempersmier et al., 2015) We herein showed that PGC1α is required for brown adipocyte differentiation in young PVASCs. Aging results in a decreased PGC1α expression in PVASCs. PGC1α‐knockdown inhibits young PVASC‐to‐brown adipocyte differentiation and accelerates neointimal hyperplasia after perivascular delivery to injured arteries. PGC1α is critical for tube formation in vitro and PVASC‐to‐endothelial cell differentiation in vivo. In addition, brown adipocyte‐specific pgc1α deficiency may be useful to demonstrate its role in vascular remodeling, and these needs further investigation in the future by utilizing transgenic models. Likely even more valuable is that PGC1α‐overexpression rescues the brown adipocyte differentiation capacity of hPVASCs from old CABG patients (average 63 years old). Accordantly, PGC1α‐overexpression improves hPVASC delivery‐induced neointimal hyperplasia in injured arteries of nude mice. CABG is widely used for the treatment of coronary heart disease and remains the most common form of cardiac surgery.(Alexander & Smith, 2016) Neointimal hyperplasia‐induced vascular restenosis is a common adverse event following CABG surgery.(Gaudino et al., 2017) We herein provide an altered potential therapy target to prevent graft restenosis, and it deserves detailed preclinical investigation in the future. In summary, our results provide direct evidence to characterize the transcriptional landscape of resident stromal cells in the PVAT. These findings further indicate the complication of PVAT in the vascular health and disease and expand the biological functions of PVAT in the vascular remodeling process. More importantly, loss of PGC1α in aged PVASCs show decreased endothelial and adipogenic differentiation, especially brown adipocyte generation and contribute to neointimal hyperplasia after vascular injury. Taken together, these provide a possibility that regulation of PGC1α‐mediated PVASC differentiation may be a therapeutic potential to prevent/treat aging‐related vascular diseases.

## 4 EXPERIMENTAL PROCEDURES

A detailed Materials and Methods section is available in the Appendix S1.

### 4.1 Mice

Wild‐type, green fluorescent protein (GFP) transgenic mice on a C57BL/6 background were obtained from The Jackson Laboratory. Nude mice on a Balb/c background were purchased from the Model Animal Research Center of Nanjing University. All animal procedures were approved in accordance with institutional guidelines established by the Committee of Ethics on Animal Experiments at Shanghai Jiao Tong University School of Medicine.

### 4.2 Cell isolation and flow cytometric analysis

Mouse PVAT was dissected from the thoracic aorta as previously described.(Chang et al., 2012) To avoid the effects of the adventitia, only lipid droplet positive tissue was collected. Adipose tissue was cut into small pieces and then digested with 0.2% collagenase (Sigma‐Aldrich) at 37°C for 45 min. The cell suspension was filtered through 70‐µm nylon cell strainer (Falcon) twice to remove tissue debris. The stromal cells were resuspended in red blood cell lysis buffer (eBioscience) for 10 min and then were maintained in 1% bovine serum albumin (BSA) in PBS containing fluorochrome‐conjugated antibodies directed against the following cell surface markers: CD45‐Alexa Fluor700, Lin‐eFluor450, Sca1‐PE‐Cy7, CD90‐PE, cKit‐APC, CD34‐FITC (eBioscience). Flow cytometry was performed on a BD flow cytometer (Verse). FCS files were exported and analyzed using FlowJo 8.3.3 software (Tree Star Inc).

### 4.3 Single‐cell RNA sequence

Primary cultured PVASCs of PVAT were digested by trypsin. Young or old cellular suspensions (3,000 cells) were loaded on a 10× Chromium instrument (10× Genomics) according to manufacturer's protocol based on the 10× GEMCode proprietary technology. The scRNA‐seq libraries were prepared using Chromium Single Cell 3’ v2 Reagent Kit (10× Genomics) according to manufacturer's protocol. Library quantification and quality assessment were performed using Qubit fluorometric assay (Invitrogen) with dsDNA HS (High Sensitivity) Assay Kit and Bioanalyzer Agilent 2,100 using a High Sensitivity DNA chip (Agilent Genomics). Indexed libraries were equimolarly pooled and sequenced on an Illumina HiSeq2500 using paired‐end 26 × 98 bp as sequencing mode. Single‐cell expression data were analyzed using the Cell Ranger Single Cell Software Suite (v1.3.1) to perform quality control, sample demultiplexing, barcode processing, and single‐cell 3′ gene counting. Sequencing reads were aligned to the UCSC hg19 transcriptome using the Cell Ranger suite with default parameters. Mean raw reads per cell were 50,000 for young and old cells, respectively. Raw Illumina sequencing data from Chromium Single Cell libraries were transformed into the file formats that are ready for downstream analysis using the Cell Ranger pipeline. R package Seurat (version 2.0) was used for single‐cell RNA‐seq data downstream analysis followed on guideline analysis with default parameters as previously described.(Butler, Hoffman, Smibert, Papalexi, & Satija, 2018).

### 4.4 Vascular injury, perivascular delivery of PVASCs, and histological analysis

The old donor or recipient mice were 18–20 months old, and young mice were 2–3 months old. Vascular injury and perivascular delivery of PVASCs were performed as previously described. (Hage et al., 2010; Psaltis et al., 2014) To induce vascular injury, mice were subjected to carotid artery ligation. Young or old mice were anesthetized with isoflurane and left carotid arteries were completely ligated just proximal to the carotid bifurcation. Meanwhile, PVASCs at passage 2 obtained from young and old GFP donor mice were resuspended in Matrigel (2 × 10^5^ in 50 μL) and injected (27 G needle) as a bleb into the perivascular tissue of the ligated carotid artery of recipient mice. Twenty‐eight days after injury, the left carotid artery was harvested and flushed with 0.01 M sodium phosphate buffer and fixed with 10% formalin for 24 hr. Vessels were then processed for paraffin embedding and cut into 4‐µm transverse sections for hematoxylin–eosin (H&E) and picrosirius red staining. Morphometric analysis was performed using Image‐Pro Plus software to assess neointimal hyperplasia by measuring the vessel and lumen area. Fibrotic staining was expressed as a percentage of stained areas (red) to the total areas examined.

### 4.5 Human PVASCs isolation and culture

Perivascular adipose tissue samples were collected from the patients who underwent surgery for CABG at Ruijin hospital. From the ascending aorta area ready for the proximal anastomosis during revascularization surgery, 2‐cm rectangular aortic PVAT specimens were harvested as rapidly as possible from the greater curvature of the aorta for further management. The clinical characteristics are shown in Table S2. The PVAT was digested to obtain stromal cells as mentioned above. The hPVASCs were cultured for flow cytometric analysis, cell differentiation, and implantation to nude mice at passage 3. This human study was approved by the Ethics Committee of Ruijin Hospital, Shanghai Jiao Tong University School of Medicine and conducted in accordance with the Declaration of Helsinki. Signed informed consent was obtained from all study participants.

### 4.6 Statistical analysis

Statistical analysis was carried out using SPSS 19 (SPSS). Comparisons of experimental groups were analyzed by Student's *t* test (two groups) or 1‐way ANOVA followed by the post hoc Dunnett's test for data with more than two groups (Levene's tests for equal variance). Dunnett's T3 test was used as post hoc test comparison for the analysis of unequal variances (Welch's and Brown–Forsythe's test). Values are represented as mean ± *SD*. The significance level was set at *p* < 0.05.

## CONFLICT OF INTEREST

None declared.

## AUTHOR CONTRIBUTIONS

P.J.G, C.C.R., and X.X.P. designed the study and wrote the manuscript. X.X.P., C.C.R., L.R.K., and Z.Z.B. conducted in vitro studies. X.X.P., C.C.R., Y.M., and Q.H.W. performed in vivo studies. H.Q.L., Y.J.S., A.Q.C., and Q.Z collected samples from patients. C.C.R, X.X.P, and X.Y.L performed statistical analyses with assistance from P.J.G, X.J.W, J.G.W, D.L.Z, and W.F.

## References

[acel12969-bib-0001] Aldiss, P. , Davies, G. , Woods, R. , Budge, H. , Sacks, H. S. , & Symonds, M. E. (2017). ‘Browning’ the cardiac and peri‐vascular adipose tissues to modulate cardiovascular risk. International Journal of Cardiology, 228, 265–274. 10.1016/j.ijcard.2016.11.074 27865196 PMC5236060

[acel12969-bib-0002] Alexander, J. H. , & Smith, P. K. (2016). Coronary‐Artery Bypass Grafting. New England Journal of Medicine, 374, 1954–1964. 10.1056/NEJMra1406944 27192673

[acel12969-bib-0003] Alt, E. U. , Senst, C. , Murthy, S. N. , Slakey, D. P. , Dupin, C. L. , Chaffin, A. E. , … Izadpanah, R. (2012). Aging alters tissue resident mesenchymal stem cell properties. Stem Cell Res, 8, 215–225. 10.1016/j.scr.2011.11.002 22265741

[acel12969-bib-0004] Antonopoulos, A. S. , Margaritis, M. , Coutinho, P. , Shirodaria, C. , Psarros, C. , Herdman, L. , … Antoniades, C. (2015). Adiponectin as a link between type 2 diabetes and vascular NADPH oxidase activity in the human arterial wall: The regulatory role of perivascular adipose tissue. Diabetes, 64, 2207–2219. 10.2337/db14-1011 25552596

[acel12969-bib-0005] Antonopoulos, A. S. , Sanna, F. , Sabharwal, N. , Thomas, S. , Oikonomou, E. K. , Herdman, L. , … Antoniades, C. (2017). Detecting human coronary inflammation by imaging perivascular fat. Science Translational Medicine, 9(398), eaal2658 10.1126/scitranslmed.aal2658 28701474

[acel12969-bib-0006] Bhumiratana, S. , Bernhard, J. C. , Alfi, D. M. , Yeager, K. , Eton, R. E. , Bova, J. , … Vunjak‐Novakovic, G. (2016). Tissue‐engineered autologous grafts for facial bone reconstruction. Science Translational Medicine, 8, 343ra383 10.1126/scitranslmed.aad5904 PMC4944852 27306665

[acel12969-bib-0007] Brown, N. K. , Zhou, Z. , Zhang, J. , Zeng, R. , Wu, J. , Eitzman, D. T. , … Chang, L. (2014). Perivascular adipose tissue in vascular function and disease: A review of current research and animal models. Arteriosclerosis, Thrombosis, and Vascular Biology, 34, 1621–1630. 10.1161/ATVBAHA.114.303029 PMC4104287 24833795

[acel12969-bib-0008] Butler, A. , Hoffman, P. , Smibert, P. , Papalexi, E. , & Satija, R. (2018). Integrating single‐cell transcriptomic data across different conditions, technologies, and species. Nature Biotechnology, 36, 411–420. 10.1038/nbt.4096 PMC6700744 29608179

[acel12969-bib-0009] Chang, L. , Villacorta, L. , Li, R. , Hamblin, M. , Xu, W. , Dou, C. , … Chen, Y. E. (2012). Loss of perivascular adipose tissue on peroxisome proliferator‐activated receptor‐gamma deletion in smooth muscle cells impairs intravascular thermoregulation and enhances atherosclerosis. Circulation, 126, 1067–1078. 22855570 10.1161/CIRCULATIONAHA.112.104489 PMC3493564

[acel12969-bib-0010] Chatterjee, T. K. , Stoll, L. L. , Denning, G. M. , Harrelson, A. , Blomkalns, A. L. , Idelman, G. , … Weintraub, N. L. (2009). Proinflammatory phenotype of perivascular adipocytes: Influence of high‐fat feeding. Circulation Research, 104, 541–549. 10.1161/CIRCRESAHA.108.182998 19122178 PMC2742882

[acel12969-bib-0011] Dempersmier, J. , Sambeat, A. , Gulyaeva, O. , Paul, S. M. , Hudak, C. S. , Raposo, H. F. , … Sul, H. S. (2015). Cold‐inducible Zfp516 activates UCP1 transcription to promote browning of white fat and development of brown fat. Molecular Cell, 57, 235–246. 10.1016/j.molcel.2014.12.005 25578880 PMC4304950

[acel12969-bib-0012] Eghbalieh, S. D. D. , Chowdhary, P. , Muto, A. , Ziegler, K. R. , Kudo, F. A. , Pimiento, J. M. , … Dardik, A. (2012). Age‐related neointimal hyperplasia is associated with monocyte infiltration after balloon angioplasty. Journals of Gerontology. Series A, Biological Sciences and Medical Sciences, 67, 109–117. 10.1093/gerona/glr190 PMC3261442 22016364

[acel12969-bib-0013] Fleenor, B. S. , Eng, J. S. , Sindler, A. L. , Pham, B. T. , Kloor, J. D. , & Seals, D. R. (2014). Superoxide signaling in perivascular adipose tissue promotes age‐related artery stiffness. Aging Cell, 13, 576–578. 10.1111/acel.12196 24341314 PMC4326900

[acel12969-bib-0014] Friederich‐Persson, M. , Nguyen Dinh Cat, A. , Persson, P. , Montezano, A. C. , & Touyz, R. M. (2017). Brown adipose tissue regulates small artery function through NADPH oxidase 4‐derived hydrogen peroxide and redox‐sensitive protein kinase G‐1 alpha. Arteriosclerosis, Thrombosis, and Vascular Biology, 37, 455–465. 10.1161/ATVBAHA.116.308659 28062507

[acel12969-bib-0015] Gaudino, M. , Antoniades, C. , Benedetto, U. , Deb, S. , Di Franco, A. , Di Giammarco, G. , … Bakaeen, F. G. (2017). Mechanisms, Consequences, and Prevention of Coronary Graft Failure. Circulation, 136, 1749–1764. 10.1161/CIRCULATIONAHA.117.027597 29084780

[acel12969-bib-0016] Gómez‐Serrano, M. , Camafeita, E. , López, J. A. , Rubio, M. A. , Bretón, I. , García‐Consuegra, I. , … Peral, B. (2017). Differential proteomic and oxidative profiles unveil dysfunctional protein import to adipocyte mitochondria in obesity‐associated aging and diabetes. Redox Biology, 11, 415–428. 10.1016/j.redox.2016.12.013 28064117 PMC5220168

[acel12969-bib-0017] Goodell, M. A. , & Rando, T. A. (2015). Stem cells and healthy aging. Science, 350, 1199–1204. 10.1126/science.aab3388 26785478

[acel12969-bib-0018] Greenstein, A. S. , Khavandi, K. , Withers, S. B. , Sonoyama, K. , Clancy, O. , Jeziorska, M. , … Heagerty, A. M. (2009). Local inflammation and hypoxia abolish the protective anticontractile properties of perivascular fat in obese patients. Circulation, 119, 1661–1670. 10.1161/CIRCULATIONAHA.108.821181 19289637

[acel12969-bib-0019] Hage, F. G. , Oparil, S. , Xing, D. , Chen, Y. F. , McCrory, M. A. , & Szalai, A. J. (2010). C‐reactive protein‐mediated vascular injury requires complement. Arteriosclerosis, Thrombosis, and Vascular Biology, 30, 1189–1195. 10.1161/ATVBAHA.110.205377 PMC2897052 20339115

[acel12969-bib-0020] Hassanpour, M. , Cheraghi, O. , Siavashi, V. , Rahbarghazi, R. , & Nouri, M. (2016). A reversal of age‐dependent proliferative capacity of endothelial progenitor cells from different species origin in in vitro condition. J Cardiovasc Thorac Res, 8, 102–106. 10.15171/jcvtr.2016.22 27777694 PMC5075357

[acel12969-bib-0021] Kiefer, F. W. , Cohen, P. , & Plutzky, J. (2012). Fifty shades of brown: Perivascular fat, thermogenesis, and atherosclerosis. Circulation, 126, 1012–1015. 10.1161/CIRCULATIONAHA.112.123521 22927471 PMC3711937

[acel12969-bib-0022] Lakatta, E. G. , & Levy, D. (2003). Arterial and cardiac aging: Major shareholders in cardiovascular disease enterprises: Part I: Aging arteries: A "set up" for vascular disease. Circulation, 107, 139–146. 10.1161/01.CIR.0000048892.83521.58 12515756

[acel12969-bib-0023] Margaritis, M. , Antonopoulos, A. S. , Digby, J. , Lee, R. , Reilly, S. , Coutinho, P. , … Antoniades, C. (2013). Interactions between vascular wall and perivascular adipose tissue reveal novel roles for adiponectin in the regulation of endothelial nitric oxide synthase function in human vessels. Circulation, 127, 2209–2221. 10.1161/CIRCULATIONAHA.112.001133 23625959

[acel12969-bib-0024] Meyer, M. R. , Fredette, N. C. , Daniel, C. , Sharma, G. , Amann, K. , Arterburn, J. B. , … Prossnitz, E. R. (2016). Obligatory role for GPER in cardiovascular aging and disease. Science Signalling, 9, ra105 10.1126/scisignal.aag0240 PMC5124501 27803283

[acel12969-bib-0025] Pierce, G. L. , Lesniewski, L. A. , Lawson, B. R. , Beske, S. D. , & Seals, D. R. (2009). Nuclear factor‐{kappa}B activation contributes to vascular endothelial dysfunction via oxidative stress in overweight/obese middle‐aged and older humans. Circulation, 119, 1284–1292. 19237660 10.1161/CIRCULATIONAHA.108.804294 PMC2810548

[acel12969-bib-0026] Psaltis, P. J. , Puranik, A. S. , Spoon, D. B. , Chue, C. D. , Hoffman, S. J. , Witt, T. A. , … Simari, R. D. (2014). Characterization of a resident population of adventitial macrophage progenitor cells in postnatal vasculature. Circulation Research, 115, 364–375. 10.1161/CIRCRESAHA.115.303299 24906644

[acel12969-bib-0027] Rajsheker, S. , Manka, D. , Blomkalns, A. L. , Chatterjee, T. K. , Stoll, L. L. , & Weintraub, N. L. (2010). Crosstalk between perivascular adipose tissue and blood vessels. Current Opinion in Pharmacology, 10, 191–196. 10.1016/j.coph.2009.11.005 20060362 PMC2843777

[acel12969-bib-0028] Rivera‐Gonzalez, G. C. , Shook, B. A. , Andrae, J. , Holtrup, B. , Bollag, K. , Betsholtz, C. , … Horsley, V. (2016). Skin adipocyte stem cell self‐renewal is regulated by a PDGFA/AKT‐signaling axis. Cell Stem Cell, 19, 738–751. 10.1016/j.stem.2016.09.002 27746098 PMC5135565

[acel12969-bib-0029] Rodriguez‐Menocal, L. , Faridi, M. H. , Martinez, L. , Shehadeh, L. A. , Duque, J. C. , Wei, Y. , … Vazquez‐Padron, R. I. (2014). Macrophage‐derived IL‐18 and increased fibrinogen deposition are age‐related inflammatory signatures of vascular remodeling. American Journal of Physiology. Heart and Circulatory Physiology, 306, H641–653. 10.1152/ajpheart.00641.2013 24414074 PMC3949070

[acel12969-bib-0030] Ruan, C.‐C. , Ge, Q. , Li, Y. , Li, X.‐D. , Chen, D.‐R. , Ji, K.‐D. , … Gao, P.‐J. (2015). Complement‐mediated macrophage polarization in perivascular adipose tissue contributes to vascular injury in deoxycorticosterone acetate‐salt mice. Arteriosclerosis, Thrombosis, and Vascular Biology, 35, 598–606. 10.1161/ATVBAHA.114.304927 25573852

[acel12969-bib-0031] Silva, F. J. , Holt, D. J. , Vargas, V. , Yockman, J. , Boudina, S. , Atkinson, D. , … Patel, A. N. (2014). Metabolically active human brown adipose tissue derived stem cells. Stem Cells, 32, 572–581. 10.1002/stem.1595 24420906

[acel12969-bib-0032] Takaoka, M. , Nagata, D. , Kihara, S. , Shimomura, I. , Kimura, Y. , Tabata, Y. , … Sata, M. (2009). Periadventitial adipose tissue plays a critical role in vascular remodeling. Circulation Research, 105, 906–911. 10.1161/CIRCRESAHA.109.199653 19762682

[acel12969-bib-0033] Wang, M. , Monticone, R. E. , & Lakatta, E. G. (2010). Arterial aging: A journey into subclinical arterial disease. Current Opinion in Nephrology and Hypertension, 19, 201–207. 10.1097/MNH.0b013e3283361c0b 20040868 PMC2943205

[acel12969-bib-0034] Withers, S. B. , Bussey, C. E. , Saxton, S. N. , Melrose, H. M. , Watkins, A. E. , & Heagerty, A. M. (2014). Mechanisms of adiponectin‐associated perivascular function in vascular disease. Arteriosclerosis, Thrombosis, and Vascular Biology, 34, 1637–1642. 10.1161/ATVBAHA.114.303031 24855062

[acel12969-bib-0035] Xiong, S. , Patrushev, N. , Forouzandeh, F. , Hilenski, L. , & Alexander, R. W. (2015). PGC‐1 alpha modulates telomere function and DNA damage in protecting against aging‐related chronic diseases. Cell Reports, 12, 1391–1399. 26299964 10.1016/j.celrep.2015.07.047 PMC4549794

[acel12969-bib-0036] Xiong, S. , Salazar, G. , Patrushev, N. , Ma, M. , Forouzandeh, F. , Hilenski, L. , & Alexander, R. W. (2013). Peroxisome proliferator‐activated receptor gamma coactivator‐1 alpha is a central negative regulator of vascular senescence. Arteriosclerosis, Thrombosis, and Vascular Biology, 33, 988–998. 10.1161/ATVBAHA.112.301019 PMC3663327 23430617

[acel12969-bib-0037] Xu, M. , Pirtskhalava, T. , Farr, J. N. , Weigand, B. M. , Palmer, A. K. , Weivoda, M. M. , … Kirkland, J. L. (2018). Senolytics improve physical function and increase lifespan in old age. Nature Medicine. 10.1038/s41591-018-0092-9 PMC6082705 29988130

