# Supplementary material for: Perivascular adipose tissue‐derived stromal cells contribute to vascular remodeling during aging
Source: Aging Cell. 2019 May 14;18(4):e12969. doi: 10.1111/acel.12969 (PMC6612678; doi:10.1111/acel.12969)

Figure S1

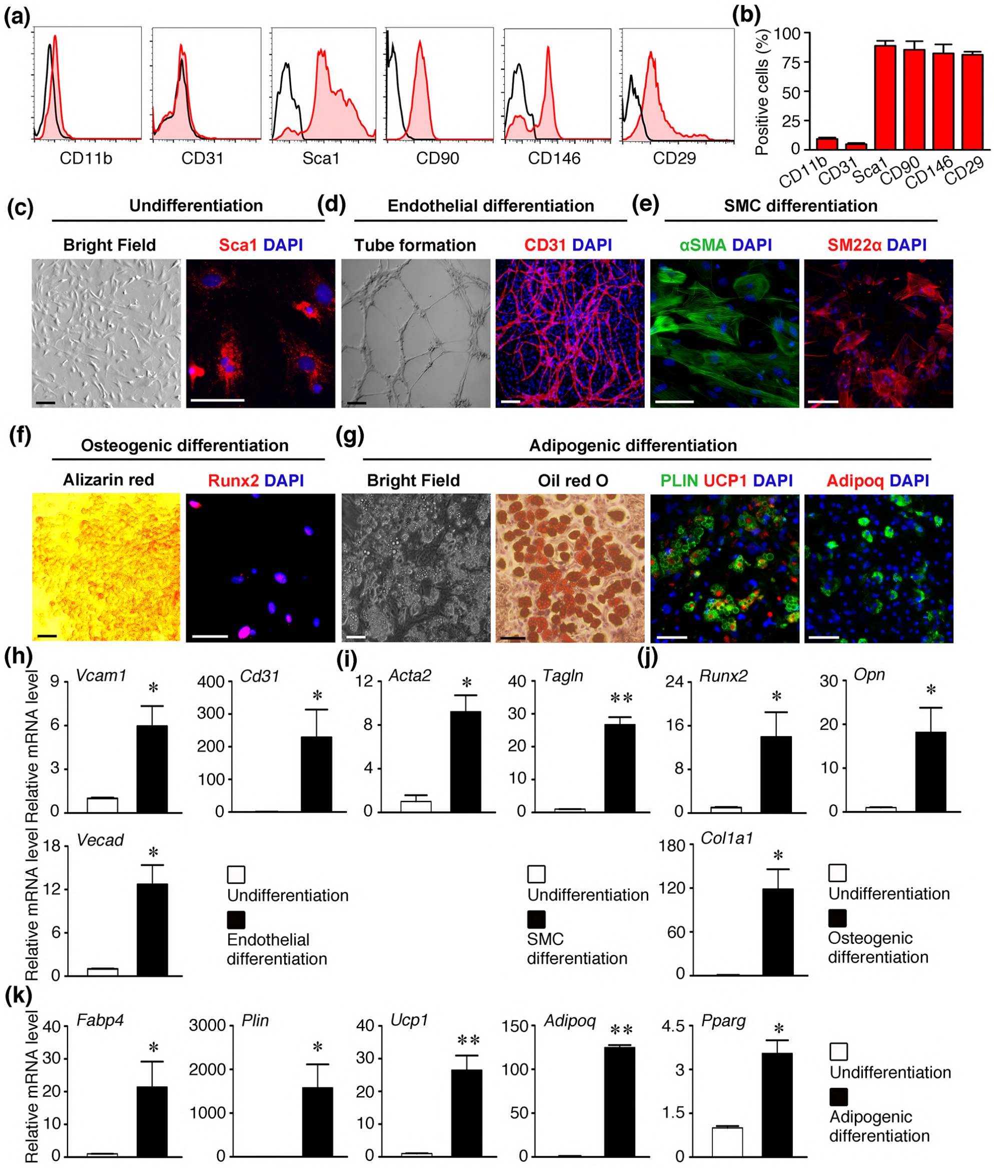
(a)
CD11b
CD31
Sca1
CD90
CD146
CD29
(b)
Positive cells (%)
100
75
50
25
0
CD11b
CD31
Sca1
CD90
CD146
CD29
(c)
Undifferentiation
Bright Field
Sca1 DAPI
(d)
Endothelial differentiation
Tube formation
CD31 DAPI
(e)
SMC differentiation
αSMA DAPI
SM22α DAPI
(f)
Osteogenic differentiation
Alizarin red
Runx2 DAPI
(g)
Adipogenic differentiation
Bright Field
Oil red O
PLIN UCP1 DAPI
Adipoq DAPI
(h)
Relative mRNA level
Vcam1
Cd31
Vecad
Undifferentiation
Endothelial differentiation
(i)
Acta2
Tagln
Undifferentiation
SMC differentiation
(j)
Runx2
Opn
Col1a1
Undifferentiation
Osteogenic differentiation
(k)
Relative mRNA level
Fabp4
Plin
Ucp1
Adipoq
Pparg
Undifferentiation
Adipogenic differentiation

Figure S2

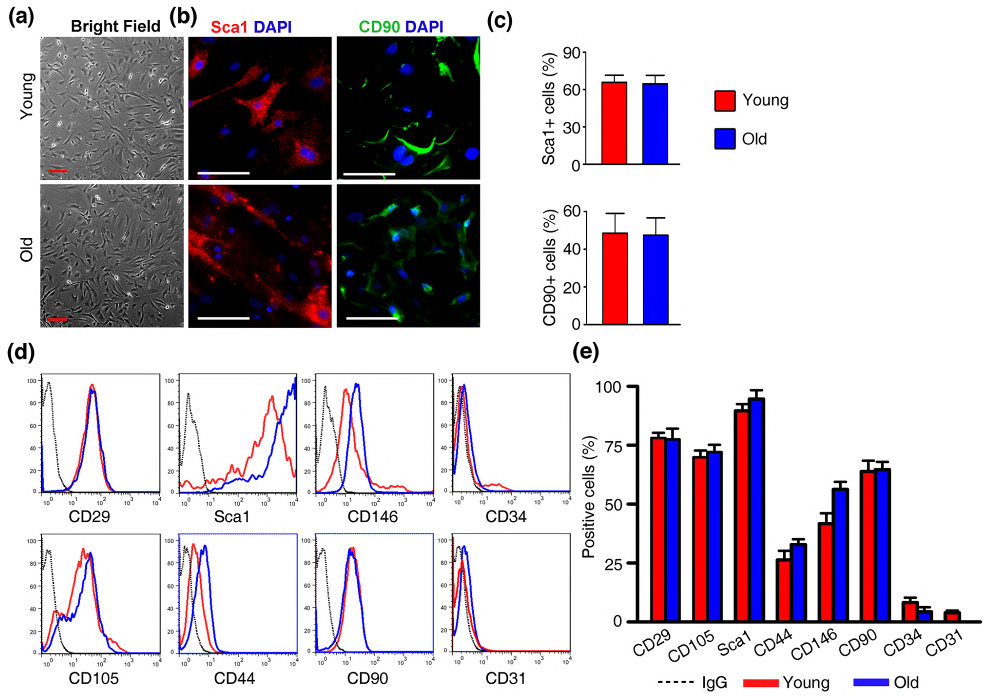

Figure S3

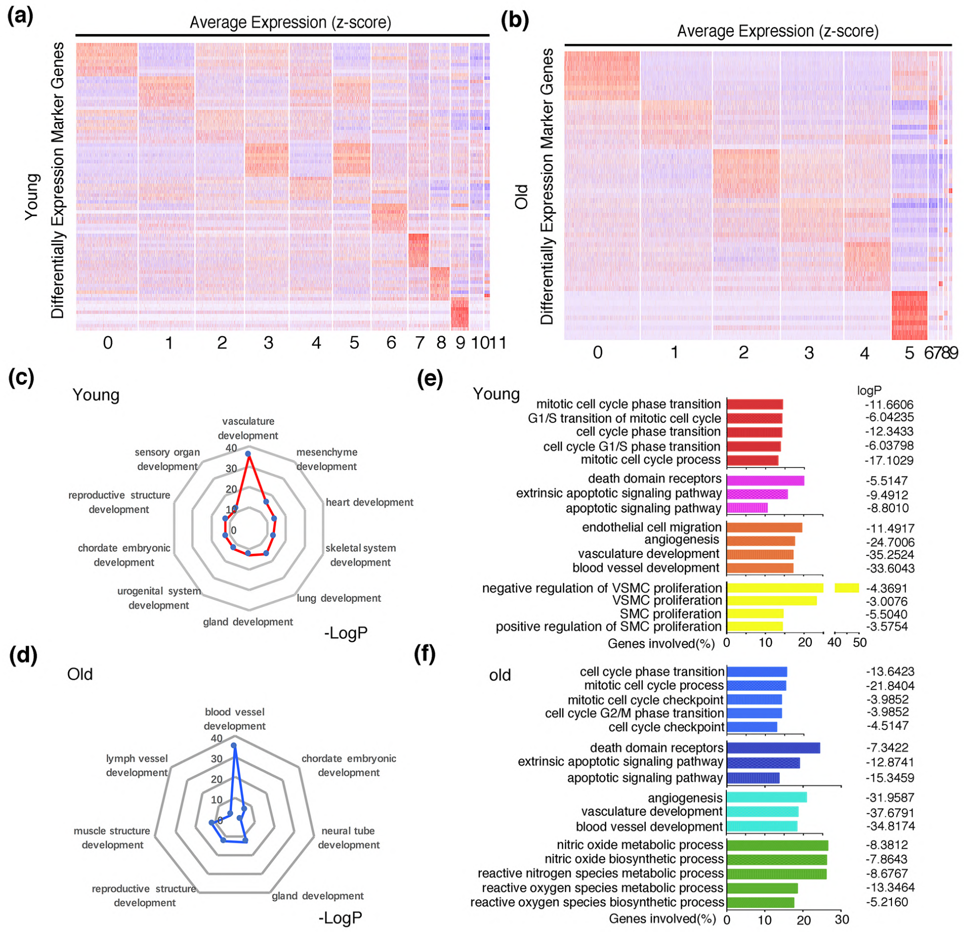

Figure S4

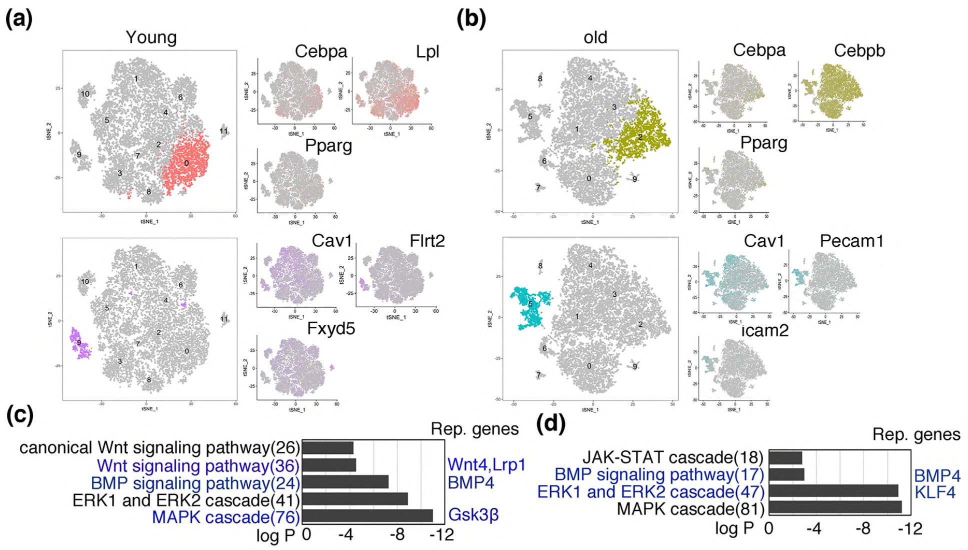

Figure S5

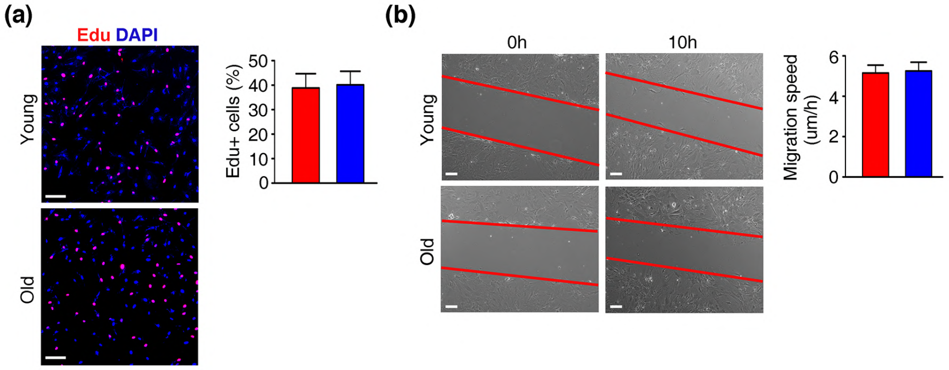

Figure S6

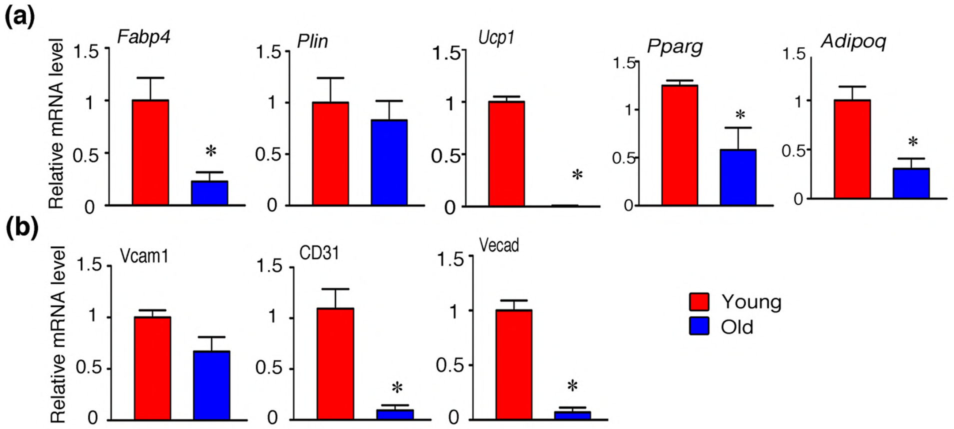

Figure S7

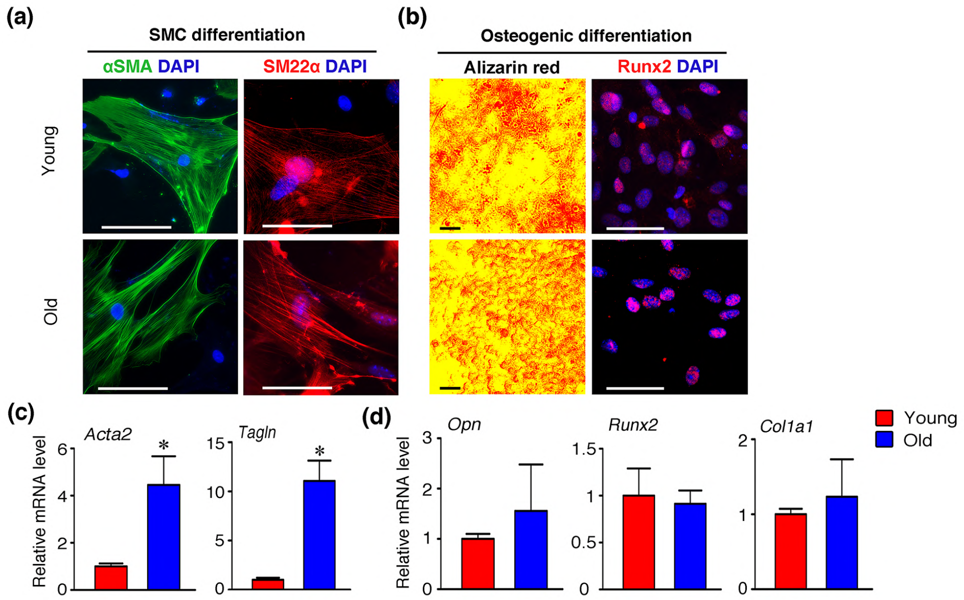

Figure S8

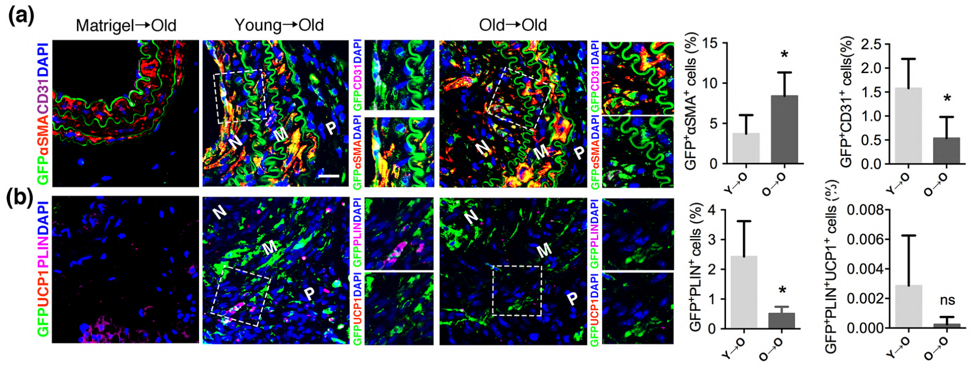

Figure S9

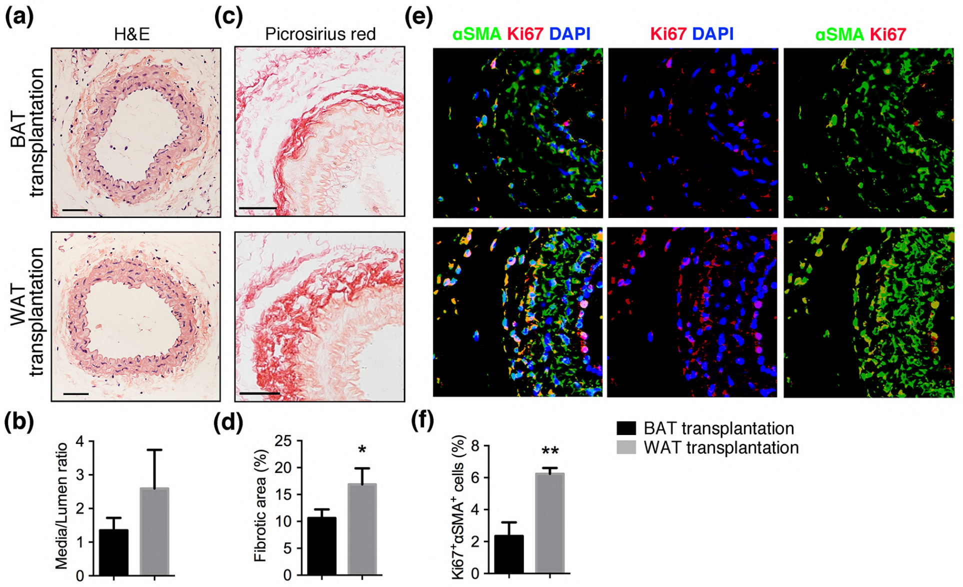

Figure S10

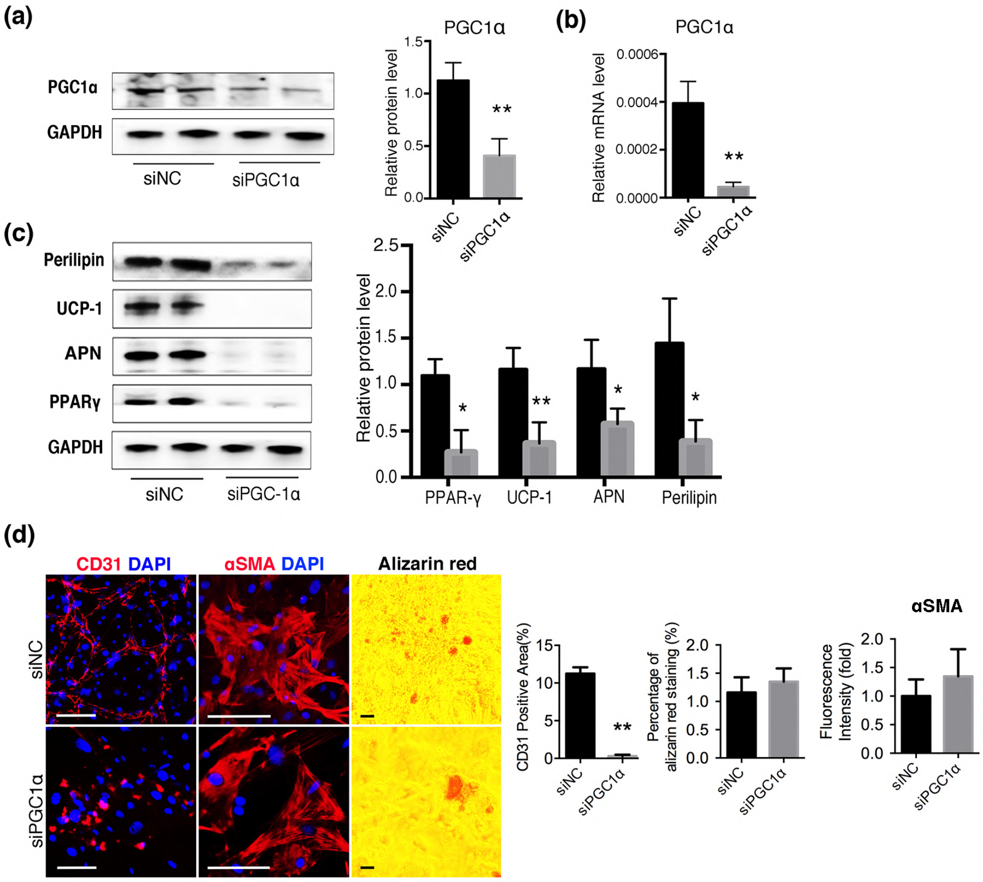

Figure S11

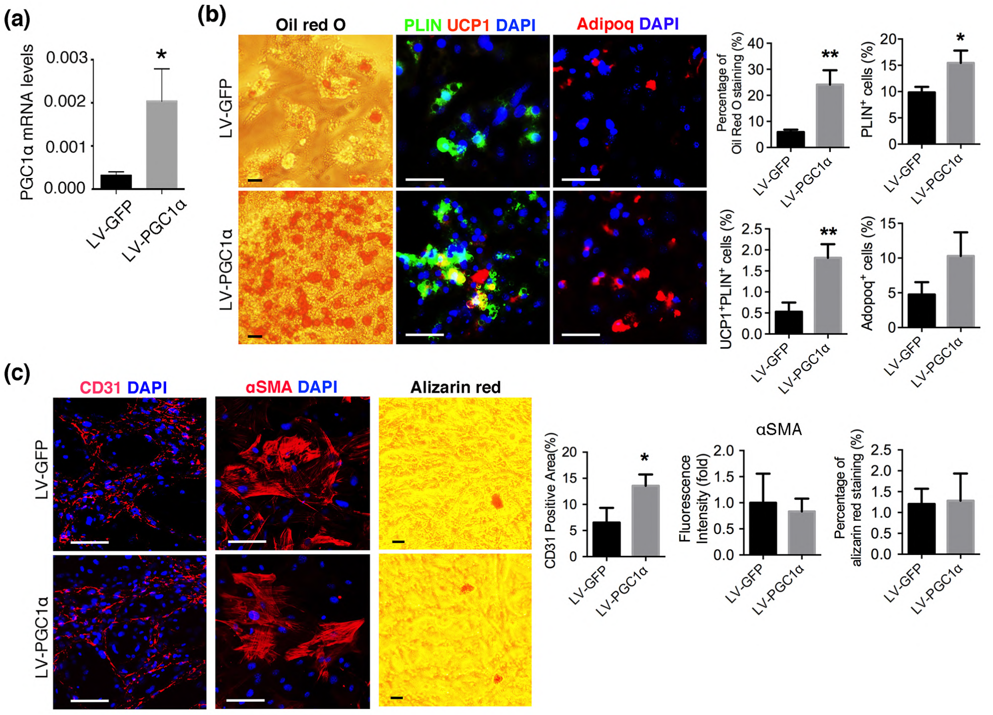

Figure S12

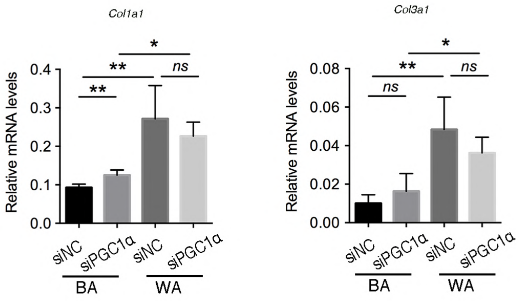

Figure S13

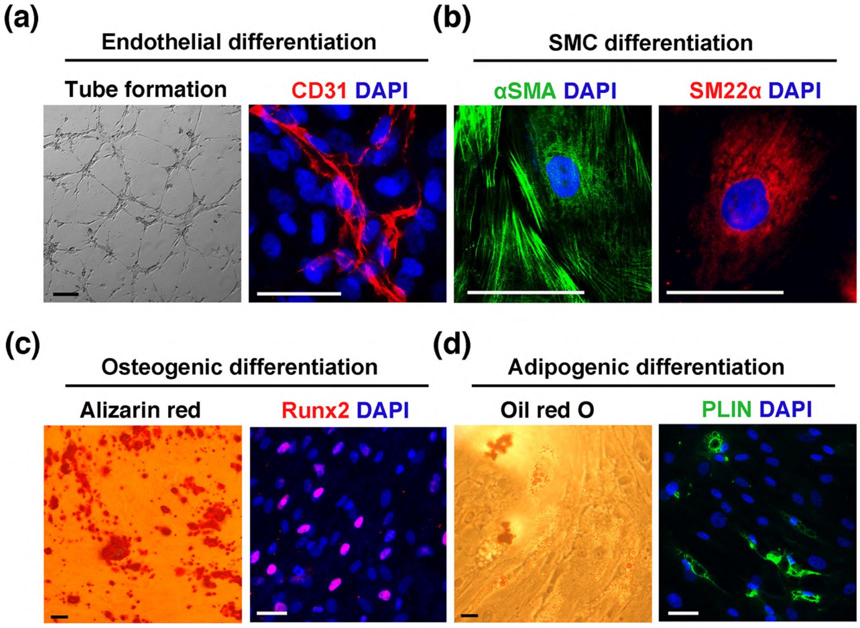

Figure S14

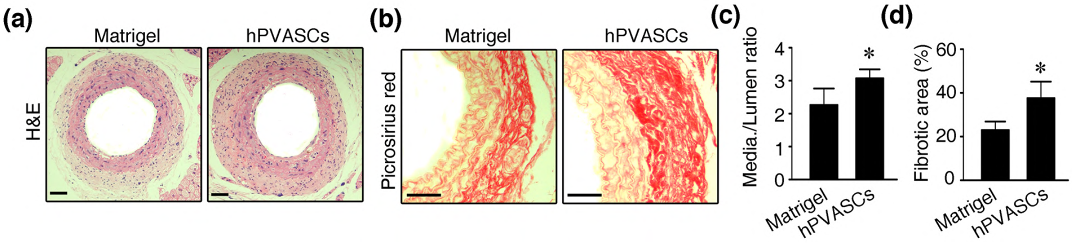

Supplement: Supplementary file 1 [file ACEL-18-e12969-s001.pdf]
