# Supplementary material for: Perivascular adipose tissue‐derived stromal cells contribute to vascular remodeling during aging
Source: Aging Cell. 2019 May 14;18(4):e12969. doi: 10.1111/acel.12969 (PMC6612678; doi:10.1111/acel.12969)
Supplement: Supplementary file 2 [file ACEL-18-e12969-s002.pdf]

Young					
Cluster	Symbol	description	gene	avg_logFC	p_val
0	Lhx8	Mus musculus LIM homeobox protein 8 (Lhx8), mRNA.	ENSMUSG00000096225	0.26198	7.60E-100
0	Cebpa	Mus musculus CCAAT/enhancer binding protein (C/EBP), alpha (Cebpa), transcript variant 1, mRNA.	ENSMUSG00000034957	0.26475	1.77E-110
0	Lpl	Mus musculus lipoprotein lipase (Lpl), mRNA.	ENSMUSG00000015568	1.14415	0.00E+00
0	Ebf1	Mus musculus early B cell factor 1 (Ebf1), transcript variant 4, mRNA.	ENSMUSG00000057098	0.66786	5.3175801277
0	Hp	Mus musculus haptoglobin (Hp), mRNA.	ENSMUSG00000031722	0.61382	8.35E-214
1	Mef2c	Mus musculus myocyte enhancer factor 2C (Mef2c), transcript variant 2, mRNA.	ENSMUSG00000005583	0.26040	3.13E-68
1	Nexn	Mus musculus nexilin (Nexn), mRNA.	ENSMUSG00000039103	0.29390	2.80E-81
1	Grem1	Mus musculus gremlin 1 (Grem1), mRNA.	ENSMUSG00000074934	0.62425	2.48E-138
1	Myh10	Mus musculus myosin, heavy polypeptide 10, non-muscle (Myh10), mRNA.	ENSMUSG00000020900	0.59513	4.89E-280
2	Cited2	Mus musculus Cbp/p300-interacting transactivator, with Glu/Asp-rich carboxy-terminal domain, 2 (Cited2), mRNA.	ENSMUSG00000039910	0.29744	1.33E-46
2	Acta2	Mus musculus actin, alpha 2, smooth muscle, aorta (Acta2), mRNA.	ENSMUSG00000035783	0.32350	4.54E-86
3	Tacc3	Mus musculus transforming, acidic coiled-coil containing protein 3 (Tacc3), mRNA.	ENSMUSG00000037313	0.26519	3.87E-112
3	Plk4	Mus musculus polo-like kinase 4 (Plk4), transcript variant 3, non-coding RNA.	ENSMUSG00000025758	0.26362	4.85E-109
3	Ckap4	Mus musculus cytoskeleton-associated protein 4 (Ckap4), mRNA.	ENSMUSG00000046841	0.30135	6.87E-102
3	Hjurp	Mus musculus Holliday junction recognition protein (Hjurp), mRNA.	ENSMUSG00000044783	0.29766	8.32E-99

3	Kpnb1	Mus musculus karyopherin (importin) beta 1 (Kpnb1), mRNA.	ENSMUSG00000001440	0.29386	2.34E-86
3	Dnmt1	Mus musculus DNA methyltransferase (cytosine-5) 1 (Dnmt1), transcript variant 4, mRNA.	ENSMUSG00000004099	0.25850	5.00E-86
4	Prrx1	Mus musculus paired related homeobox 1 (Prrx1), transcript variant 3, mRNA.	ENSMUSG00000026586	0.28441	2.09E-63
4	Igfbp7	Mus musculus insulin-like growth factor binding protein 7 (Igfbp7), transcript variant 2, mRNA.	ENSMUSG00000036256	0.27509	1.53E-53
4	Bdnf	Mus musculus brain derived neurotrophic factor (Bdnf), transcript variant 9, mRNA.	ENSMUSG00000048482	0.25086	3.83E-31
4	Fgf18	Mus musculus fibroblast growth factor 18 (Fgf18), transcript variant 2, non-coding RNA.	ENSMUSG00000057967	0.27862	3.52E-88
4	Myo10	Mus musculus myosin X (Myo10), mRNA.	ENSMUSG00000022272	0.28834	8.19E-66
5	Mki67	Mus musculus antigen identified by monoclonal antibody Ki 67 (Mki67), mRNA.	ENSMUSG00000031004	0.47829	1.78E-229
5	Cenpf	Mus musculus centromere protein F (Cenpf), mRNA.	ENSMUSG00000026605	0.60253	1.15E-206
5	Top2a	Mus musculus topoisomerase (DNA) II alpha (Top2a), mRNA.	ENSMUSG00000020914	0.78160	4.25E-254
5	Ube2c	Mus musculus ubiquitin-conjugating enzyme E2C (Ube2c), mRNA.	ENSMUSG00000001403	0.78796	7.81E-254
5	Nusap1	Mus musculus nucleolar and spindle associated protein 1 (Nusap1), transcript variant 2, mRNA.	ENSMUSG00000027306	0.42571	7.65E-169
6	Col3a1	Mus musculus collagen, type III, alpha 1 (Col3a1), mRNA.	ENSMUSG00000026043	0.59689	1.90E-173
6	Dcn	Mus musculus decorin (Dcn), transcript variant 2, mRNA.	ENSMUSG00000019929	0.44687	3.56E-113
6	Pde1a	Mus musculus phosphodiesterase 1A, calmodulin-dependent (Pde1a), transcript variant 9, mRNA.	ENSMUSG00000059173	0.28770	2.36E-69

6	Ptn	Mus musculus pleiotrophin (Ptn), mRNA.	ENSMUSG00000029838	0.80996	2.11E-98
6	Tcf21	Mus musculus transcription factor 21 (Tcf21), mRNA.	ENSMUSG00000045680	0.36601	4.78E-154
6	Hand2	Mus musculus heart and neural crest derivatives expressed transcript 2 (Hand2), mRNA.	ENSMUSG00000038193	0.30865	2.28E-183
6	Gata4	Mus musculus GATA binding protein 4 (Gata4), mRNA.	ENSMUSG00000021944	0.31126	5.16E-257
7	Notch2	Mus musculus notch 2 (Notch2), mRNA.	ENSMUSG00000027878	0.79387	1.54E-292
7	Rhoa	Mus musculus ras homolog gene family, member A (Rhoa), mRNA.	ENSMUSG00000007815	0.34815	3.91E-80
7	Twf1	Mus musculus twinfilin, actin-binding protein, homolog 1 (Drosophila) (Twf1), mRNA.	ENSMUSG00000022451	0.26326	6.21E-89
7	Skil	Mus musculus SKI-like (Skil), transcript variant 3, mRNA.	ENSMUSG00000027660	0.29733	1.86E-77
7	Gsk3b	Mus musculus glycogen synthase kinase 3 beta (Gsk3b), mRNA.	ENSMUSG00000022812	0.34847	6.90E-90
7	Actr2	Mus musculus ARP2 actin-related protein 2 (Actr2), mRNA.	ENSMUSG00000020152	0.31246	2.36E-91
8	Jag1	Mus musculus jagged 1 (Jag1), mRNA.	ENSMUSG00000027276	0.28918	8.07E-49
8	Creb3l1	Mus musculus cAMP responsive element binding protein 3-like 1 (Creb3l1), mRNA.	ENSMUSG00000027230	0.37742	5.27E-64
8	Cthrc1	Mus musculus collagen triple helix repeat containing 1 (Cthrc1), mRNA.	ENSMUSG00000054196	1.10510	4.99E-287
8	Serpinh1	Mus musculus serine (or cysteine) peptidase inhibitor, clade H, member 1 (Serpinh1), transcript variant 4, mRNA.	ENSMUSG00000070436	0.26624	9.38E-60
8	Col11a1	Mus musculus collagen, type XI, alpha 1 (Col11a1), mRNA.	ENSMUSG00000027966	0.27100	1.35E-30
8	Ctgf	Mus musculus connective tissue growth factor (Ctgf), mRNA.	ENSMUSG00000019997	0.49481	2.12E-51

8	Tgfb1	Mus musculus transforming growth factor, beta 1 (Tgfb1), mRNA.	ENSMUSG00000002603	0.69456	9.88E-229
8	Chst11	Mus musculus carbohydrate sulfotransferase 11 (Chst11), mRNA.	ENSMUSG00000034612	0.36929	3.15E-94
8	Loxl2	Mus musculus lysyl oxidase-like 2 (Loxl2), mRNA.	ENSMUSG00000034205	0.45689	4.77E-74
8	Creb3l2	Mus musculus cAMP responsive element binding protein 3-like 2 (Creb3l2), mRNA.	ENSMUSG00000038648	0.36300	1.23E-67
9	F11r	Mus musculus F11 receptor (F11r), mRNA.	ENSMUSG00000038235	0.48822	3.68E-191
9	Cxadr	Mus musculus coxsackie virus and adenovirus receptor (Cxadr), transcript variant 3, mRNA.	ENSMUSG00000022865	1.58338	0.00E+00
9	Flrt2	Mus musculus fibronectin leucine rich transmembrane protein 2 (Flrt2), mRNA.	ENSMUSG00000047414	1.27363	0.00E+00

Old					
cluster	Symbol	description	gene	avg_logFC	p_val
0	Mki67	Mus musculus antigen identified by monoclonal antibody Ki 67 (Mki67), mRNA.	ENSMUSG00000031004	0.74663	0.00E+00
0	Cenpf	Mus musculus centromere protein F (Cenpf), mRNA.	ENSMUSG00000026605	0.81072	0.00E+00
0	Top2a	Mus musculus topoisomerase (DNA) II alpha (Top2a), mRNA.	ENSMUSG00000020914	1.19446	0.00E+00
0	Ube2c	Mus musculus ubiquitin-conjugating enzyme E2C (Ube2c), mRNA.	ENSMUSG00000001403	1.18105	0.00E+00
0	Nusap1	Mus musculus nucleolar and spindle associated protein 1 (Nusap1), transcript variant 2, mRNA.	ENSMUSG00000027306	0.59208	0.00E+00
1	Lgals1	Mus musculus lectin, galactose binding, soluble 1 (Lgals1), mRNA.	ENSMUSG00000068220	0.25414	0.00E+00
1	Actn1	Mus musculus actinin, alpha 1 (Actn1), mRNA.	ENSMUSG00000015143	0.37822	3.02E-175
1	Hmgb2	Mus musculus high mobility group box 2 (Hmgb2), mRNA.	ENSMUSG00000054717	1.03654	0.00E+00

1	Hmgb1	Mus musculus high mobility group box 1 (Hmgb1), mRNA.	ENSMUSG00000066551	0.57101	0.00E+00
2	Cebpa	Mus musculus CCAAT/enhancer binding protein (C/EBP), alpha (Cebpa), transcript variant 1, mRNA.	ENSMUSG00000034957	0.42571	8.07E-229
2	Rgs2	Mus musculus regulator of G-protein signaling 2 (Rgs2), mRNA.	ENSMUSG00000026360	0.30334	1.52E-82
2	Sfrp1	Mus musculus secreted frizzled-related protein 1 (Sfrp1), mRNA.	ENSMUSG00000031548	0.96541	0.00E+00
2	Sfrp2	Mus musculus secreted frizzled-related protein 2 (Sfrp2), mRNA.	ENSMUSG00000027996	0.55916	2.77E-198
2	Fabp4	Mus musculus fatty acid binding protein 4, adipocyte (Fabp4), mRNA.	ENSMUSG00000062515	1.22104	2.2232954062
2	Lpl	Mus musculus lipoprotein lipase (Lpl), mRNA.	ENSMUSG00000015568	1.01431	0.00E+00
2	Hp	Mus musculus haptoglobin (Hp), mRNA.	ENSMUSG00000031722	1.61710	0.00E+00
3	Col1a2	Mus musculus collagen, type I, alpha 2 (Col1a2), mRNA.	ENSMUSG00000029661	0.27974	5.52E-170
3	Col3a1	Mus musculus collagen, type III, alpha 1 (Col3a1), mRNA.	ENSMUSG00000026043	0.50657	4.16E-218
3	Col1a1	Mus musculus collagen, type I, alpha 1 (Col1a1), mRNA.	ENSMUSG00000001506	0.26966	2.84E-153
3	Enc1	Mus musculus ectodermal-neural cortex 1 (Enc1), mRNA.	ENSMUSG00000041773	0.31844	3.87E-95
3	Fn1	Mus musculus fibronectin 1 (Fn1), transcript variant 7, mRNA.	ENSMUSG00000026193	0.31211	2.34E-183
4	Sox9	Mus musculus SRY (sex determining region Y)-box 9 (Sox9), mRNA.	ENSMUSG00000000567	0.41919	2.23E-122
4	Limd1	Mus musculus LIM domains containing 1 (Limd1), mRNA.	ENSMUSG00000025239	0.26191	1.73E-87

4	Vegfa	Mus musculus vascular endothelial growth factor A (Vegfa), transcript variant 6, mRNA.	ENSMUSG00000023951	0.31902	5.44E-88
4	Spp1	Mus musculus secreted phosphoprotein 1 (Spp1), transcript variant 5, mRNA.	ENSMUSG00000029304	1.01621	6.05E-137
4	Nppc	Mus musculus natriuretic peptide type C (Nppc), mRNA.	ENSMUSG00000026241	0.47073	1.58E-109
4	Lmna	Mus musculus lamin A (Lmna), transcript variant 3, mRNA.	ENSMUSG00000028063	0.87051	0.00E+00
4	Jund	Mus musculus jun D proto-oncogene (Jund), transcript variant 1, mRNA.	ENSMUSG00000071076	0.62423	3.00E-303
4	Junb	Mus musculus jun B proto-oncogene (Junb), mRNA.	ENSMUSG00000052837	0.32090	2.59E-78
4	Igfbp5	Mus musculus insulin-like growth factor binding protein 5 (Igfbp5), mRNA.	ENSMUSG00000026185	0.95163	1.62E-108
4	Fgfr1	Mus musculus fibroblast growth factor receptor 1 (Fgfr1), transcript variant 3, mRNA.	ENSMUSG00000031565	0.33086	8.61E-107
4	Fgf2	Mus musculus fibroblast growth factor 2 (Fgf2), mRNA.	ENSMUSG00000037225	0.38631	2.23E-86
4	Twist2	Mus musculus twist basic helix-loop-helix transcription factor 2 (Twist2), mRNA.	ENSMUSG00000007805	0.29773	5.48E-83
5	Icam2	Mus musculus intercellular adhesion molecule 2 (Icam2), mRNA.	ENSMUSG00000001029	0.48381	0.00E+00
5	Pecam1	Mus musculus platelet/endothelial cell adhesion molecule 1 (Pecam1), transcript variant 4, mRNA.	ENSMUSG00000020717	1.17269	0.00E+00
5	Eng	Mus musculus endoglin (Eng), transcript variant 3, mRNA.	ENSMUSG00000026814	1.16418	0.00E+00
5	Cdh5	Mus musculus cadherin 5 (Cdh5), mRNA.	ENSMUSG00000031871	1.49569	0.00E+00
5	Emcn	Mus musculus endomucin (Emcn), transcript variant 2, mRNA.	ENSMUSG00000054690	2.00324	0.00E+00

5	Ctla2b	Mus musculus cytotoxic T lymphocyte-associated protein 2 beta (Ctla2b), transcript variant 2, mRNA.	ENSMUSG00000074874	2.03499	0.00E+00

Table S1. Aaverage expression for representative marker genes and cell-type classification of the main clusters from young and old data set
