# Supplementary material for: Perivascular adipose tissue‐derived stromal cells contribute to vascular remodeling during aging
Source: Aging Cell. 2019 May 14;18(4):e12969. doi: 10.1111/acel.12969 (PMC6612678; doi:10.1111/acel.12969)
Supplement: Supplementary file 3 [file ACEL-18-e12969-s003.pdf]

No.	Age	Sex	BMI	BP (mmHg)	Dyslipidemia	Diabetes	Cardiac function	Diagnosis	Medication
1	55	M	24.1	138/64	TG (2.22) HDL(0.84)	+	II	Aortic dissection	-
2	71	M	24.1	127/55	-	+	II-III	CHD triple vessle lesion	ACEI, Insulin
3	53	M	21.1	160/62	-	-	III	Aneurysm of ascending aorta	-
4	44	M	22.5	133/82	TG (4.73) TC(5.03)	-	-	CHD triple vessle lesion	β-blocker
5	79	F	21.5	131/64	TC(5.51)	+	II	CHD triple vessle lesion, Coronary artery aneurysm	-
6	47	M	27.5	133/76	TG (3.63) HDL(0.74)	-	II	CHD triple vessle lesion	β-blocker,Statin, Aspirin, Clopidogel
7	61	F	21.9	112/78	TC(5.16)	-	-	Congenital heart disease, Aortic insufficiency	Anticoagulant
8	75	F	19.1	115/67	-	-	-	CHD	Diuretic, Anticoagul ant
9	73	F	25.9	139/72	-	+	II	CHD triple vessle lesion, Varicose veins of the left lower extremity	spirolactone
10	62	M	19.6	140/82	TG(1.85)	-	II	CHD, Postcholecystectomy	-
11	74	M	20.8	113/77	HDL(0.99)	+	III	CHD	Diuretic， spirolactone， Metformin

12	58	F	21.5	120/77	TC(6.53)<br>HDL(0.88)	-	II- III	CHD	Diuretic， spirolactone
13	65	M	28.1	153/83	TG(2.50)<br>TC(6.23)	+	III	CHD, Peripheral vascular disease	Diuretic， spirolactone，<br>Metformin

Table S2 The clinical characteristics of human samples.

BMI, Body mass index; BP, Blood pressure; M, Male; F, Female; TG, Triglyceride; TC: Total cholesterol; HDL, High density lipoprotein, CHD, Coronary heart disease.
