# Supplementary material for: Perivascular adipose tissue‐derived stromal cells contribute to vascular remodeling during aging
Source: Aging Cell. 2019 May 14;18(4):e12969. doi: 10.1111/acel.12969 (PMC6612678; doi:10.1111/acel.12969)
Supplement: Supplementary file 4 [file ACEL-18-e12969-s004.docx]

**Supplementary Materials**

Supplementary Figure Legend

Figure S1. The multiple differentiation capacities of perivascular adipose tissue-derived stromal cells (PVASCs). (a) Flow cytometric analysis of PVASCs showed positive markers of mesenchymal stem cell and negative markers of macrophage and endothelial cell. (b) Quantitative analysis of flow cytometry data. (c-g) The cultured PVASCs showed differentiation capacities towards endothelial cells (d), smooth muscle cells (SMCs) (e), osteoblasts (f) and adipocytes (g) by specific staining. Scale bar 50 μm. (h-k) The differentiation were determined by qPCR analysis of cell specific markers. *p<0.05, **p<0.001 vs undifferentiation, n=4 independent experiments.

Figure S2. Aging does not affect immunophenotypic profiles of perivascular adipose tissue-derived stromal cells (PVASCs). (a) The surface morphology of PVASCs obtained from young or old mice. Scale bar 50μm. (b, c) The expression of mesenchymal stem cells marker Sca1 and CD90 was measured by immunostaining. (d, e) The surface markers of young and old PVASCs were measured by flow cytometry. n=4 independent experiments.

Figure S3.Gene ontology analysis of young or old scRNA-seq data set. (a,b) Heatmap of differentially expressed cell-type marker genes across each of the main cluster of young or old. (c,d) Gene ontology analysis of biological process of young or old scRNA-seq data set was shown on radar map. (e,f) Several gene ontology terms and their corresponding logP values (listed on the right).

Figure S4 (a,b) Heatmap expression of selected genes that are commonly expressed in adipogenic lineage cells (Cebpa, Lpl, Pparg and Cebpb) or endothelial lineage cells (Cav1, Flrt2, Fxyd5, Pecam1 and Icam2) were plotted onto scRNA-seq clusters. (c,d) Gene ontology analysis of signaling pathway associtated with adipogenic differentiation. The number of genes is shown in parentheses.

Figure S5. Aging does not affect proliferation and migration of PVASCs. (a) The proliferation of young and old PVASCs was measured by Edu staining. Red indicated positive staining for Edu. (b) The migration of young and old PVASCs was measured by wound-healing migration assay. The right panel showed the average migration speed 10h after scraping. Scale bar 50 μm. n=4 independent experiments.

Figure S6 The difference of specific genes between young and old differentiated perivascular adipose tissue-derived stromal cells (PVASCs). (a,b) The adipogenic (a) and endothelial (b) differentiation were determined by qPCR of cell specific markers. *p<0.05 vs Young. n=4 independent experiments.

Figure S7 The SMC and osteogenic differentiation capacities of perivascular adipose tissue-derived stromal cells (PVASCs) were altered during aging. (a,b) The osteogenic and SMC differentiation were determined by specific staining in the young and old PVASCs. Scale bar 50 μm. (c,d) The SMC (c) and osteogenic (d) differentiation were determined by qPCR of cell specific markers. *p<0.05 vs Young. n=4 independent experiments.

Figure S8 The vascular microenvironment aging restrained PVASC-to-brown adipocytes differentiation. The carotid arteries sections were co-stained for GFP and CD31, GFP and SMA, GFP and PLIN, GFP and UCP1 28 days after young or old PVASCs implanted to old mice. Scale bar 50 μm. n=5 per group.

Figure S9 BAT transplantation improved neointimal formation after vascular injury. (a-d) H&E and picrosirius red-stained sections of carotid arteries, and quantitative analysis of medium-lumen ratio and arterial fibrotic area of injured * p< 0.05. Scale bar 50 μm. n=5 per group. (e,f) The carotid arteries sections were co-stained for αSMA and Ki67.

Figure S10. The effects of PGC1α on differentiation capacities of peivascular adipose tissue-derived stromal cells (PVASCs). (a,b) The expression of PGC1α was determined by western blot(a) and qPCR(b). (c) The adipogenic differentiaion was determined by western blot of specific markers. The right panels showed the quantitive analysis of relative protein levels. *p<0.05, **p<0.001 vs negative control cells. n=4 independent experiments. (d) The endothelial (CD31), SMC (αSMA) and osteogenic (Alizarin red) differentiation was determined by specific staining in the young PVASCs infected with lentivirus-mediated PGC1α (siPGC1α) or negative control (siNC). Scal bar 50μm.

Figure S11 Overexpression of PGC1α rejuvenated the adipogenic differentiation capacity of old PVASCs. (a) The expression of PGC1α was detected by qPCR. *p<0.05, vs negative control. (b) The adipogenic differentiation was determined by specific staining in the old PVASCs infected with lentivirus-mediated PGC1α on negative controls. (c) endothelial (CD31), SMC (αSMA) and osteogenic (Alizarin red) differentiation was determined by specific staining. Scal bar 50μm. n=4 independent experiments.

Figure S12. Knockdown of PGC1α in PVASCs promoted expression of Col1a1 and Col3a1 in the cultured VSMC. *p<0.05, **p<0.01. n=4 independent experiments.

Figure S13. The multiple differentiation capacities of human perivascular adipose tissue-derived stromal cells (hPVASCs). The cultured hPVASCs showed differentiation capacities towards endothelial cells (a), smooth muscle cells (SMCs) (b), osteoblasts (c) and adipocytes (d) by specific staining. Scale bar 50 μm. n=4 independent experiments.

Figure S14. Peri-carotid arterial delivery of human perivascular adipose tissue-derived stromal cells (hPVASCs) accelerates neointima hyperplasia in nude mice after ligation injury. (a, b) Representative light micrographs of H&E and picrosirius red-stained sections of carotid arteries. (c, d) Quantitive analysis of medium-lumen ratio and arterial fibrotic area of post-ligation Scale bar 50 μm. * p < 0.05 versus Materigel. n=5 per group.

Materials and Methods

**Mice**

Wild type, green fluorescent protein (GFP) transgenic mice on a C57BL/6 background were obtained from The Jackson Laboratory. Nude mice on a Balb/c background were purchased from Model Animal Research Center of Nanjing University. All animal procedures were approved in accordance with institutional guidelines established by the Committee of Ethics on Animal Experiments at Shanghai Jiao Tong University School of Medicine.

**Cell Isolation and Flow Cytometric Analysis**

Mouse PVAT was dissected from thoracic aorta as previously described.[^3^](#_ENREF_3) To avoid the effects of adventitia, only lipid droplet positive tissue was collected. Adipose tissue were cut into small pieces, which then digested with 0.2% collagenase (Sigma-Aldrich, St. Louis, MO, USA) at 37°C for 45 min. The cell suspension was filtered through 70-um Nylon cell strainer (Falcon, Duham, NC, USA) for twice to remove tissue debris. The stromal vascular fraction (SVF) cells were resuspended in red blood cell lysis buffer (eBioscience, San Diego, CA, USA) for 10 min and then were maintained in 1% bovine serum albumin (BSA) in PBS containing fluorochrome-conjugated antibodies directed against the following cell surface markers: CD45-Alexa Fluor700, Lin-eFluor450, Sca1-PE-Cy7, CD90-PE, cKit-APC, CD34-FITC (eBioscience). Flow cytometry was performed on a BD flow cytometer (Verse). FCS files were exported and analyzed using FlowJo 8.3.3 software (Tree Star Inc., Ashland, OR, USA).

**Immunofluorescent Staining**

Paraffin sections were first deparaffinized and rehydrated. After boiling in 10 mM sodium citrate buffer to unmask antigens. For cultured cells, glass coverslips with cells were fixed in metganol. Then the slides were blocked in buffer containing 5% normal goat serum, incubated with primary antibodies at 4°C overnight, followed by incubation with fluorochrome-conjugated secondary antibodies (Invitrogen). Cellular nuclei were stained with DAPI (1:1000, Invitrogen). Primary antibodies used were as followings: PLIN (1:100, Abcam), Sca1 (1:100, BD Biosciences), GFP (1:200, Abcam), CD31 (1:100, BD Biosciences), αSMA (1:300, Sigma), SM22α (1:200, Abcam), Runx2 (1:200, Abcam), UCP1 (1:100, Abcam), Adipoq (1:200, R&D), Ki67 (1:200, Thermo Scientific).

**Perivascular Adipose Tissue-derived Stem Cells (PVASCs) Culture and Differentiation**

SVF cells obtained from PVAT were maintained in DMEM/F12 medium supplemented with 10%FBS and 1% penicillin/streptomycin at 37°C with 5% CO_2_. Medium is changed the day after seeding and then every second day. All PVASCs used for subsequent experiments were prepared from 2-3 passage.

For endothelial cell differentiation, cells were cultured for 8 days in basal medium supplemented with 50 ng/mL VEGF and 2 ng/mL bFGF. The medium was changed every 2 days. For tube formation, 500 μL Matrigel was coated on each well of a 12-well plate and incubated for 1 h at 37°C to allow the gel to solidify. Next, 2 × 105 ASCs were seeded in base culture medium. After incubation for 12 h, the cells were visualized using a light microscope (Zeiss).

For SMC differentiation, cultured cells at 80% confluence were switched to medium with 5-Azacytidine (10μM). After 24 hours, cells were cultured for 7-10 days in medium with 2% horse serum, without FBS. The medium was changed every 3 days.

For osteogenic differentiation, cells were cultured for 14-21 days in osteogenic differentiation medium which consisted in basal medium supplemented with 50 µg/mL L-ascorbic acid, 10 mM glycerophosphate, and 100 nM dexamethasone. The medium was changed every 3 days.

For white adipogenic differentiation，cultured cells at 70% confluence were switched to adipogenic differentiation medium which consisted in DMEM/F12 supplemented with 10% FBS, 100 U/ml penicillin, 100 mg/ml streptomycin, Insulin ( 10μg/ml), dexamethasone (1 μM), IBMX (0.5mM). After 48 hours, IBMX and dexamethasone were removed from the medium and differentiation was extended for 6-8 days until adipocytes matured. The medium was changed every 2 days.

For brown adipogenic differentiation, cultured cells at 70% confluence were switched to adipogenic differentiation medium which consisted in DMEM／F12 supplemented with 10% FBS, 100 U/ml penicillin, 100 mg/ml streptomycin , Insulin ( 10μg/ml), dexamethasone (1 μM), IBMX (0.5mM), Rosiglitazone (1 μM) and triiodothyronine (50nM) . After 48 hours, IBMX and dexamethasone were removed from the medium and differentiation was extended for 6-8 days until adipocytes matured. The medium was changed every 2 days.

**Single-cell RNA-sequence (scRNA-seq)**

Primary cultured SVF cells of PVAT were digested by trypsin. Young or old cellular suspensions (3000 cells) were loaded on a 10X Chromium instrument (10X Genomics) according to manufacturer’s protocol based on the 10X GEMCode proprietary technology. Cellular suspensions (3000 cells) were loaded on a 10X Chromium instrument (10X Genomics) according to manufacturer’s protocol based on the 10X GEMCode proprietary technology. The scRNA-seq libraries were prepared using Chromium Single Cell 3’ v2 Reagent Kit (10X Genomics) according to manufacturer’s protocol. Library quantification and quality assessment was performed using Qubit fluorometric assay (Invitrogen) with dsDNA HS (High Sensitivity) Assay Kit and Bioanalyzer Agilent 2100 using a High Sensitivity DNA chip (Agilent Genomics). Indexed libraries were equimolarly pooled and sequenced on an Illumina HiSeq2500 using paired-end 26x98bp as sequencing mode. Single-cell expression data was analyzed using the Cell Ranger Single Cell Software Suite (v1.3.1) to perform quality control, sample de-multiplexing, barcode processing, and single-cell 3’ gene counting). Sequencing reads were aligned to the UCSC hg19 transcriptome using the Cell Ranger suite with default parameters. Mean raw reads per cell were 50000 for young and old cells respectively. Raw Illumina sequencing data from Chromium Single Cell libraries was transformed into the file formats that are ready for downstream analysis using Cell Ranger pipeline. R package Seurat (Version 2.0) was used single-cell RNA-seq data down-stream analysis followed on guideline analysis with default parameters as previously described.

**CDNA Microarray**

Total RNA was isolated and purified immediately using an RNeasy® Plus Mini Kit (Qiagen, Germany), as per manufacturer's instructions. RNA was amplified and labeled by Low Input Quick Amp Labeling Kit, One-Color (Agilent technologies, Santa Clara, CA, US), following the manufacturer’s instructions. Labeled cRNA were purified by RNeasy mini kit (QIAGEN). Each slide was hybridized with 1.65 μg Cy3-labeled cRNA using Gene Expression Hybridization Kit (Agilent technologies) in Hybridization Oven (Agilent technologies), according to the manufacturer’s instructions. After 17 hours hybridization, slides were washed in staining dishes (Thermo Shandon, Waltham, MA, US) with Gene Expression Wash Buffer Kit (Agilent technologies), followed the manufacturer’s instructions. For Data acquisition, Slides were scanned by Agilent Microarray Scannerwith default settings, Dye channel: Green, Scan resolution=3μm, PMT 100%, 20bit. Data were extracted with Feature Extraction software 10.7 (Agilent technologies). Raw data were normalized by Quantile algorithm, limma packages in R.

**Quantitative RT-PCR Analysis**

Total RNA was extracted from cultured cells using TRIzol (Invitrogen) followed by chloroform extraction according to the manufacturer’s protocol. Aliquots of total RNA were reverse transcribed into single-stranded cDNA by incubation with moloney murine leukemia virus reverse transcriptase (Promega). Real-time qRT-PCR was performed with SYBR Premix Ex Taq kits with ROX (TaKaRa) according to manufacturer’s instructions. Signals were detected on an ABI PRISM 7900 machine (Applied Biosystems). β-actin was used as a standard reference. Reactions were done at 95 °C for 30 sec followed by 40 cycles of 95 °C for 5 sec, 60 °C for 30 sec. Sequences of primers used in this study are provided in the followings: Vcam1, forward (5'-TGC CGA GCT AAA TTA CAC ATT G-3'), reverse (5'-CCT TGT GGA GGG ATG TAC AGA-3'); Vecad, forward (5'-CAA TGA CAA CTT CCC CGT CT-3'), reverse (5'-TCG TTA CGT TTG GGG TCT GT-3'); Cd31, forward (5'-ATG ACC CAG CAA CAT TCA CA-3'), reverse (5'- CAC AGA GCA CCG AAT ACC A-3'); Acta2, forward (5'- CTG ACA GAG GCA CCA TGA A-3'), reverse (5'- AGA GGC ATA GAG GGA CAG CA-3'); Tagln, forward (5'- CAA CAA GGG TCC ATC CTA CGG-3'), reverse (5'- ATC TGG GCG GCC TAC ATC A-3'); Runx2, forward (5'- CGG TCT CCT TCA GGA TGG T-3'), reverse (5'- GCT TCC GTC AGC GTC AAC A-3'); Col1a1, forward (5'-AAC CCG AGG TAT GCT TGA TCT-3'), reverse (5'-CCA GTT CTT CAT TGC ATT GC-3'); Opn forward (5'- CTC CTG GCT GAA TTC TGA GG-3'), reverse (5'-TGC CAG AAT CAG TCA CTT TCA-3'); Fabp4, forward (5'-GAT GAA ATC ACC GCA GAC GAC A-3'), reverse (5'-ATT GTG GTC GAC TTT CCA TCC C-3'); Plin, forward (5'-GTG CAA TGC CTA TGA GAA GGG TGT AC-3'), reverse (5'- GTA GAG ATG GTG CCC TTC AGT TCA GA-3'); Ucp1, forward (5'-AGG CTT CCA GTA CCA TTA GGT-3'), reverse (5'-CTG AGT GAG GCA AAG CTG ATT T-3'); Adipoq, forward (5'-GAT GGC AGA GAT GGC ACT CC-3'), reverse (5'-CTT GCC AGT GCT GCC GTC AT-3'); Pparg, forward (5'- TTA GAT GAC AGT GAC TTG GC-3'), reverse (5'-TCT TCT GGA GCA CCT TGG-3'); Actin, forward (5'- CTA AGG CCA ACC GTG AAA AGA T-3'), reverse (5'-GGG ACA GCA CAG CCT GGA T-3'.)

**Vascular Injury, Perivascular Delivery of PVASCs and Histological Analysis**

The old donor or recipient mice were 18-20 months old and young mice were 2-3 months old. Vascular injury and perivascular delivery of PVASCs were performed as previously described.[^1^](#_ENREF_1)^,^ [^2^](#_ENREF_2) To induce vascular injury, mice were subjected to carotid artery ligation. Ten-week-old mice were anesthetized with isofluorane and left carotid arteries were completely ligated just proximal to the carotid bifurcation. Meanwhile, PVASCs at passage 2 obtained from young and old GFP donor mice were resuspended in Matrigel (2x10^5^ in 50 μL) and injected (27 G needle) as a bleb into the perivascular tissue of the ligated carotid artery of recipient mice. Twenty-eight days after injury, left carotid artery was harvested and flushed with 0.01 M sodium phosphate buffer and fixed with 10% formalin for 24h. Vessels were then processed for paraffin embedding, and cut into 4-µm transverse sections for hematoxylin-eosin (H&E) and picrosirius red staining. Morphometric analysis was performed using Image-Pro Plus software to assess neointimal hyperplasia by measuring the vessel and lumen area. Fibrotic staining was expressed as a percentage of stained areas (red) to the total areas examined.

**Western Blot Analysis**

Protein lysate from tissues and cells was collected and fractionated by SDS–PAGE (10% gel) and then transferred to PVDF membranes. Membranes were incubated with primary antibodies including anti-Perilipin (Abcam, ab61682), anti-UCP-1 (Abcam, ab23841), anti-APN (R&D, AF1119), anti-PGC1α (Santa Cruz, sc-13067) and anti-PPARγ (Santa Cruz, sc-7273) overnight at 4 °C. Subsequently, the membranes washed 3 times were incubated with secondary HPR-conjugated antibodies for 2 hours at room temperature. The protein signals were detected using electrochemiluminescence (ECL) technology and quantified by imaging software Image J (Rawak Software, Inc. Germany).

**PVASC-derived Adipocyte and SMC Co-culture**

Primary rat aortic SMCs were isolated and cultured as previously described.[^4^](#_ENREF_4) Briefly, the aortic media from 8-week-old Sprague Dawley rat was aseptically dissected and digested in DMEM containing collagenase and trypsin. Isolated cells were matained in DMEM containing 10% FBS and were used at passage 4-6. PVASC-derived white adipocytes (PVASC-WAT) or brown adipocytes (PVASC-BAT) and SMCs coculture was performed by using transwell chamber. PVASCs were loaded in the lower compartment of the chamber and differentiated into white or brown adipocytes. SMCs were loaded in the upper chamber until the adipogenic differentiation accomplished. Afer 24 h incubation, the SMCs proliferation on the upper surface was measured by performing Edu assay.

**Recombinant Lentivirus**

Lentivirus (LV) expressing short-hairpin RNA (shRNA) targeting mouse PGC1α (siPGC1α) and control scramble shRNA (siNC), as well as lentivirus encoding human PGC1α (LV-PGC1α), GFP (LV-Con) and GFP-Luciferase were produced and purified by GeneChem (Shanghai, China). PGC1α shRNA sequence: ACT ATT GAG CGA ACC TTA A, no-target control shRNA sequence: TTC TCC GAA CGT GTC ACG T. The viruses were used to infect cells in the presence of Polybrene. Forty-eight hours later, primary cultured PVASCs were lysed with Trizol reagent or RIPA buffer, and the expression of PGC1α was confirmed by qPCR and Western blot analysis. These GFP positive hPVASCs were used for perivascular delivery to injuried arteries.

**Human PVASCs (hPVASCs) Isolation and Culture**

Perivascular adipose tissue (PVAT) samples were collected from the patients who underwent surgery for CABG at Ruijin hospital. From the ascending aorta area ready for the proximal anastomosis during revascularization surgery, 2-cm rectangular aortic PVAT specimens were harvested as rapidly as possible from the greater curvature of the aorta for further management. The clinical characteristics were showed in Table S1. The PVAT was digested for obtaining SVF cells as mentioned above. The hPVASCs were cultured for flow cytometric analysis, cell differentiation and implantation to nude mice at passage 3. This human study was approved by the Ethics Committee of Ruijin Hospital, Shanghai Jiao Tong University School of Medicine, and conducted in accordance with the Declaration of Helsinki. Signed informed consent was obtained from all study participants.

**In vivo Bio-luminescence Imaging**

The hPVASCs for in vivo implantation were transfected with GFP-luciferase expressed lentivirus (Genechem, Shanghai, China), then the GFP positive cells were resuspended in Matrigel (2x10^5^ in 50 μL) for perivascular delivery to ligated arteries of nude mice. Twenty eight days later, for in vivo imaging, luciferin was delivered by ip injection (20 mg for a 20 g nude mouse). Twenty minutes later, bio-luminescence imaging was acquired by IVIS Spectrum CT system (PerkinElmer) according to manufacturer’s instructions. For quantitative analysis, Region of Interest (ROI) tools were used to measure the bio-luminescence activity.

**Statistical Analysis**

Statistical analysis was carried out using SPSS 19 (SPSS). Comparisons of experimental groups were analyzed by Student’s t test (two groups) or 1-way ANOVA followed by the post-hoc Dunnett’s test for data with more than two groups (Levene’s tests for equal variance). Dunnett’s T3 test was used as post-hoc test comparison for the analysis of unequal variances (Welch’s and Brown-Forsythe’s test). Values are represented as mean ± SD. The significance level was set at P < 0.05.

**References**

1. Hage FG, Oparil S, Xing D, Chen YF, McCrory MA, Szalai AJ. C-reactive protein-mediated vascular injury requires complement. *Arterioscler Thromb Vasc Biol*. 2010;30:1189-1195.

2. Psaltis PJ, Puranik AS, Spoon DB, Chue CD, Hoffman SJ, Witt TA, Delacroix S, Kleppe LS, Mueske CS, Pan S, Gulati R, Simari RD. Characterization of a resident population of adventitial macrophage progenitor cells in postnatal vasculature. *Circ Res*. 2014;115:364-375.

3. Chang L, Villacorta L, Li R, Hamblin M, Xu W, Dou C, Zhang J, Wu J, Zeng R, Chen YE. Loss of perivascular adipose tissue on peroxisome proliferator-activated receptor-gamma deletion in smooth muscle cells impairs intravascular thermoregulation and enhances atherosclerosis. *Circulation*. 2012;126:1067-1078.

4. Majesky MW, Horita H, Ostriker A, Lu S, Regan JN, Bagchi A, Dong XR, Poczobutt J, Nemenoff RA, Weiser-Evans MC. Differentiated smooth muscle cells generate a subpopulation of resident vascular progenitor cells in the adventitia regulated by klf4. *Circ Res*. 2017;120:296-311.
